# P-Selectin mediates targeting of a self-assembling phototherapeutic nanovehicle enclosing dipyridamole for managing thromboses

**DOI:** 10.1186/s12951-023-02018-7

**Published:** 2023-08-08

**Authors:** Chia-Hung Liu, Pei-Ru Jheng, Lekha Rethi, Chandraiah Godugu, Ching Yi Lee, Yan-Ting Chen, Hieu Trung Nguyen, Er-Yuan Chuang

**Affiliations:** 1 Department of Urology, School of Medicine, College of Medicine, Taipei Medical University, Taipei, 11031 Taiwan; 2 TMU Research Center of Urology and Kidney, Taipei Medical University, Taipei, 11031 Taiwan; 3 Department of Urology, Shuang Ho Hospital, Taipei Medical University, New Taipei City, 11031 Taiwan; 4 Graduate Institute of Biomedical Materials and Tissue Engineering, International Ph.D. Program in Biomedical Engineering Graduate Institute of Biomedical Optomechatronics, Research Center of Biomedical Device, Innovation Entrepreneurship Education Center, College of Interdisciplinary Studies, Taipei Medical University, Taipei, 11031 Taiwan; 5 National Institute of Pharmaceutical Education and Research (NIPER) Hyderabad, Hyderabad, India; 6 Department of Neurosurgery, Chang Gung Memorial Hospital Linkou Main Branch and School of Medicine, College of Medicine, Chang Gung University, Taoyuan, 33305 Taiwan; 7 Department of Orthopedics and Trauma, Faculty of Medicine, University of Medicine and Pharmacy at Ho Chi Minh city, Ho Chi Minh City, 700000 Viet Nam; 8 Cell Physiology and Molecular Image Research Center, Wan Fang Hospital, Taipei Medical University, Taipei, 11696 Taiwan; 9 Precision Medicine and Translational Cancer Research Center, Taipei Medical University Hospital, Taipei, 11031 Taiwan

**Keywords:** P-selectin, Controlled release, Co-assembly, Phototherapy, Nano-assembly, Antiplatelet, Thromboembolism therapy

## Abstract

Thrombotic vascular disorders, specifically thromboembolisms, have a significant detrimental effect on public health. Despite the numerous thrombolytic and antithrombotic drugs available, their efficacy in penetrating thrombus formations is limited, and they carry a high risk of promoting bleeding. Consequently, the current medication dosage protocols are inadequate for preventing thrombus formation, and higher doses are necessary to achieve sufficient prevention. By integrating phototherapy with antithrombotic therapy, this study addresses difficulties related to thrombus-targeted drug delivery. We developed self-assembling nanoparticles (NPs) through the optimization of a co-assembly engineering process. These NPs, called DIP-FU-PPy NPs, consist of polypyrrole (PPy), dipyridamole (DIP), and P-selectin-targeted fucoidan (FU) and are designed to be delivered directly to thrombi. DIP-FU-PPy NPs are proposed to offer various potentials, encompassing drug-loading capability, targeted accumulation in thrombus sites, near-infrared (NIR) photothermal-enhanced thrombus management with therapeutic efficacy, and prevention of rethrombosis. As predicted, DIP-FU-PPy NPs prevented thrombus recurrence and emitted visible fluorescence signals during thrombus clot penetration with no adverse effects. Our co-delivery nano-platform is a simple and versatile solution for NIR-phototherapeutic multimodal thrombus control.

**Supplementary Information:**

The online version contains supplementary material available at 10.1186/s12951-023-02018-7.

## Introduction

Thrombotic vascular diseases, such as myocardial infarction, stroke, and vascular thromboembolisms, are severe and often fatal conditions that have global impacts [1–3]. These diseases result from the formation of pathological thrombi, which obstruct blood flow and cause vascular occlusion leading to ischemic injury to vital organs such as the heart, brain, and lungs [4]. As a result, there is a pressing need to develop new and effective approaches for theranostic interventions that are safe and efficient for antithrombotic clinical therapy. Anticoagulant, fibrinolytic, and antiplatelet substances are regularly used as antithrombotic medications in clinics [5]. US Food and Drug Administration-approved dipyridamole (DIP) shows potent antiplatelet activity and inhibits platelet aggregation in whole blood [6, 7]. Unfortunately, current antithrombotic substances exhibit unsatisfactory therapeutic outcomes with the possibility of hemorrhaging because of poor biodistribution, lower penetration into thrombi, and a limited therapeutic window [8, 9]. Thus, it is vital to efficiently deliver thrombolytic drugs to thrombi with minimal adverse side effects for clinical applications [10].

The discovery of a P-selectin-dependent mechanism in thrombus development has highlighted interconnections among inflammation, coagulation, and thrombosis at sites of vessel injury [11, 12]. Inflammation plays a crucial role in the early stages of endothelial cell injury and the progression of vascular thrombi [13]. One of the most extensively used techniques employs the topical application of ferric chloride (FeCl3) onto a vessel of a mouse or rat to form a thrombus [14, 15]. Notably, FeCl3 produces a thrombus through maximum oxidative stress with the production of free radicals, resulting in endothelial cell destruction and lipid peroxidation following occlusive thrombus generation. The deposition of fibrin or fibrinogen in the vascular region (wall) to form a clot also occurs when using this FeCl3 induction method [16]. P-Selectin is highly expressed in growing thrombi that develop in vessels [17] and is a promising biological target for treating thrombi in various species.

In addition to medicinal treatments, photothermal agents are being explored for near-infrared (IR; NIR) thrombus imaging and hyperthermic thrombolytic effects [18]. It was indicated that an increase in temperature in thrombotic organs efficiently relaxes fibrin clots; this utilizes hyperthermic thrombolysis and enables the triggered release of antithrombotic medications in thrombi [10]. An anticlotting effect can be achieved through a photothermal ablation approach, as previously described [19]. Animals were administered a nano-gold (Au)-based photoablation agent and subsequently exposed to NIR [19]. Remarkably, a remote noninvasive hyperthermic effect was an effective antithrombotic agent with diminished side effects, including unfavorable or life-threatening complications [19]. Accordingly, synergistic nano-photothermal treatment can reduce the risk of bleeding and provide an opportunity for safe thrombus treatment. Among NIR-absorbing nanomaterials, polypyrrole (PPy) nanocarriers are of special importance as enhanced photothermal agents, which might be an alternative for Au nanoparticle (NP) vehicles for localized photothermal therapy (PTT). This is attributable to their suitable biocompatibility, good photothermal conversion efficiency, and notable photostability [20]. However, even with an expedited diagnosis, triggered release of drugs, and accurately identified therapy, NIR-photothermal involvement is typically insufficient to eradicate thrombi due to the lack of a lesion-homing ability to precisely cause the photothermal agents to accumulate at and penetrate thrombus clots.

Combination treatment has revealed extraordinary advantages for treating thrombi to deal with the insufficient therapeutic effects of both NIR-photothermal thrombolysis and antithrombotic medications [21]. Nevertheless, it remains difficult to precisely co-deliver multiple therapeutics to thrombi [22]. Given the growth of nanomedicines, nano-drug delivery systems (nano-DDSs) [23] have revealed notable advantages in delivering antithrombotic medicines, including: (i) enhancing the physicochemical features of antithrombotic medications, (ii) understanding site-specific delivery, and (iii) decreasing the risk of bleeding. Multiple theranostic substances can be co-loaded into a single nano-system [24]. Despite the numerous benefits of co-delivering nano-DDSs, co-encapsulation of multiple therapeutic agents in traditional nanocarriers is ineffective due to low drug-loading efficiencies, a lack of fine-tuning of undesirable dosage ratios, inadequate colloidal stability, and probable leakage of medicines [25]. Furthermore, the possible off-target risk of nanocarriers has been extensively considered a problem obstructing the direct medical translation of nanomedicines [26]. Hence, there is an urgent need to create innovative co-delivery nano-systems with high efficiency, simple preparation, and minimal toxicity.

**Scheme 1:**
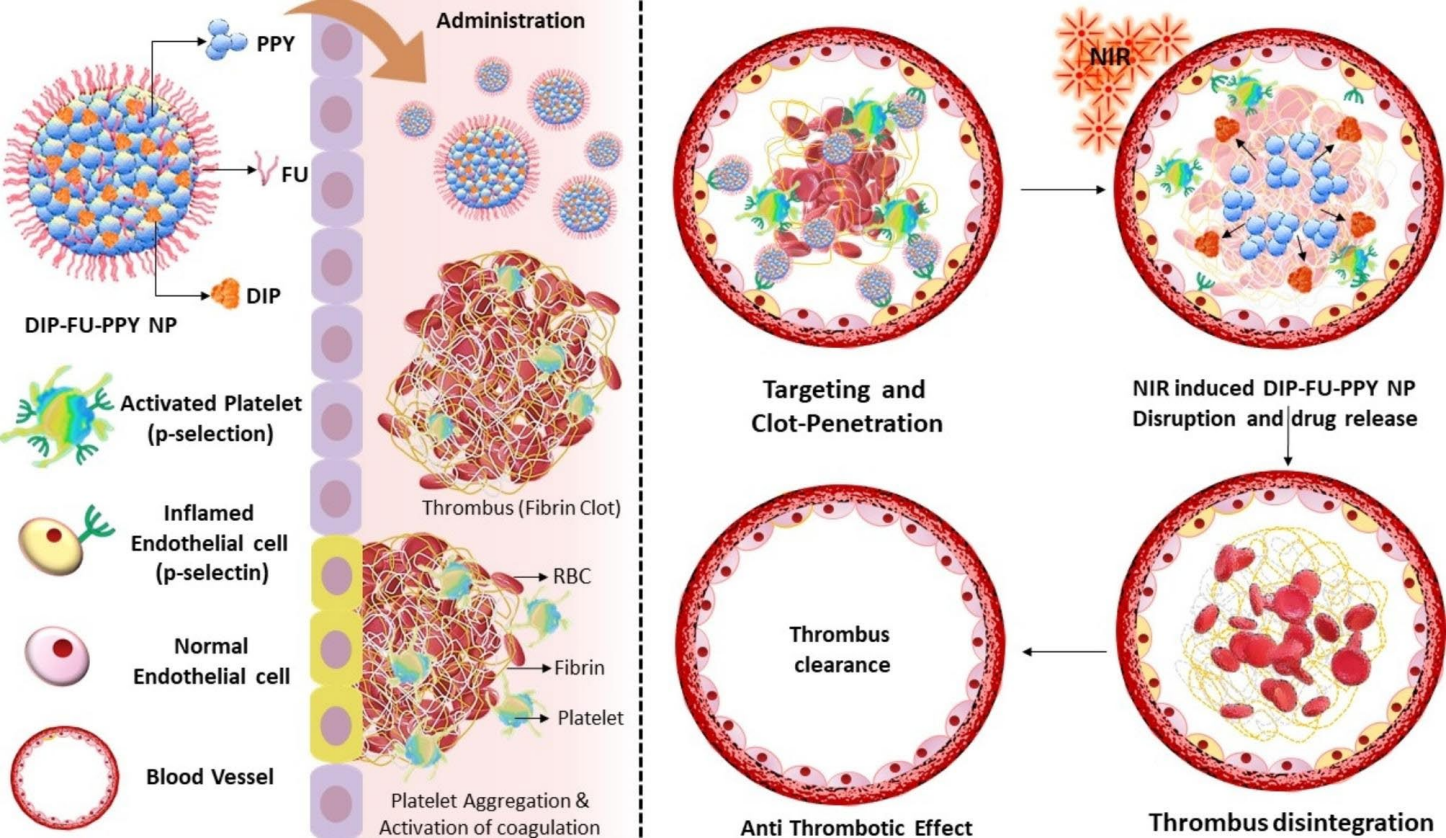
Schematic illustration depicting P-selectin-targeted fucoidan-decorated self-assembled nano-vehicle (photothermally responsive polypyrrole (PPy) probes and antithrombotic dipyridamole (DIP) combined with fucoidan (FU)) for thromboembolism therapy

Herein, we hypothesized that an accurate combination of an antiplatelet medicine and photothermal probes could be a potential approach for thrombus management. Moreover, antiplatelet medications could inhibit thrombi by reducing platelet aggregation and bioactivation, which helps avoid secondary embolisms from thrombus fragments. To test our hypothesis, a carrier assembled using hydrophobic DIP and the hydrophobic conductive polymer, PPy [27–30], was intricately engineered. The intermolecular forces and interactions were anticipated to drive the co-assembly of DIP and PPy. To improve the colloidal stability, biodistribution, and delivery performance and endow the nano-assembly with thrombus-targeting capacity, amphiphilic fucoidan (FU, a P-selectin-targeting polysaccharide) [23] was used to decorate the shell of these NPs resulting in a meticulously crafted nano-vehicle (DIP-FU-PPy NPs). We expected P-selectin-targeted DIP-FU-PPy NPs to exhibit high lesion site-specific accumulation, deep thrombus penetration, efficient NIR-photothermal conversion, and effective antithrombotic outcomes (Scheme 1).

## Materials and methods

### Materials

The pyrrole (Py) monomer and FeCl_3_-6H_2_O were purchased from Sigma-Aldrich (St. Louis, MO, USA). Fucoidan (FU) from *Laminaria japonica* (MW: 80 kDa, ester sulfate: 34%) was acquired from NOVA Pharma (Baltimore, MD, USA). Bovine aortic endothelial cells (BAECs) were harvested from primary bovine serum and cultured in cell medium (containing 10% fetal bovine serum (FBS)). Chemicals or consumables for cell culture were obtained from Life Technologies (Carlsbad, CA, USA). Other reagents or chemicals were of analytical grade and were purchased from Sigma-Aldrich.

#### Nanomaterials’ optimal formulation and physicochemical properties

To optimize the FU-PPy NPs, FU (4 mg in 1 mL of deionized (DI) water, pH 0.8) was mixed with different amounts of Py (80 mg). To these mixtures, a corresponding amount of FeCl_3_-6H_2_O (0, 0.7, 0.9, 1.1, and 1.3 mg/mL) was added and allowed to sit for approximately 15 min before subsequent particle-size measurements were made. To encapsulate DIP into the FU-PPy NPs, different amounts of DIP (0.4, 0.8, 1.2, 1.6, 2.0, 2.4, 2.8, 3.2, and 3.6 mg) were first dispersed in DI water (1 mL, pH 0.8) under magnetic stirring. Next, Py (80 mg), FU (4 mg), and FeCl_3_-6H_2_O (0.9 mg/mL) were added to the above solutions with stirring until a black product had formed (after approximately 15 min). The nanomaterials were purified through dialysis using a dialysis bag (MW: 100 kDa) to remove unpurified chemicals and other ferric chloride ions. To choose the optimal nano-formulation, the size distribution and drug-loading efficiency were examined. The optimal formulation (DIP-FU-PPy NP) was determined by identifying a suitable drug-loading capacity and particle size [31]. The concentration of the fabricated PPy-based nano-formulations was adjusted to 0.6 mg/mL by centrifugation and oven drying (at 37 °C) to calculate an accurate dry weight of the NPs. The mean NP size, zeta potential, and polydispersity index (PDI) of the PPy-based samples were tested in phosphate-buffered saline (PBS, pH 7.4) or PBS plus 10% FBS (pH 7.4) using dynamic light scattering (DLS; Zetasizer, Malvern Instruments, Worcestershire, UK), whereas the morphology of the nano-formulations was studied by transmission electron microscopy (TEM, HT7700 Hitachi, Tokyo, Japan). Gray values of the TEM data were measured using ImageJ software (National Institute of Health, Bethesda, MD, USA). Spectrophotometric data were measured using an ultraviolet-visible (UV-Vis) spectrophotometer (EPOCH2, BioTek, Winooski, VT, USA). The concentration of FU was determined using a methylene blue colorimetric assay at an absorbance of 663 nm [32]. To assess colloidal stability, DLS diameters of the NPs were measured in PBS or PBS plus 10% FBS every 24 h at 37 °C for 7 days. Morphological changes in NPs with NIR exposure (808 nm, 5 min, 2.0 W/cm^2^) were characterized by TEM and DLS. To avoid any influence of multiple scattering or issues from light affecting the sample, the solutions were sufficiently diluted to a particle concentration of approximately 0.1 vol% for DLS measurements.

#### In vitro drug release study

The drug-loading efficiency (LE) and drug-loading content (LC) are vital parameters of nanomedicines. The LE reflects the utilization of drugs in the feed during the nanomedicine-formulation procedure, whereas the LC reflects the mass ratio of drugs to nanomedicines. The optical behavior, LE, and release behavior of DIP were assessed using spectrophotometric methods [31]. The LE and LC of DIP-FU-PPy NPs were measured by blending unloaded DIP in the supernatant, and the optical density (OD) was then measured at 430 nm using a PerkinElmer EnSpire 2300 multimode plate reader (PerkinElmer, USA). The DIP LC (%) and LE (%) of the DIP-FU-PPy NPs were calculated based on the following equations: LC (%) = (mass of DIP in NPs/total mass of NPs) × 100%; and LE (%) = (mass of DIP in NPs/initial amount of feed DIP) × 100%.

The DIP release behaviors were examined by packaging DIP-FU-PPy NPs in a dialysis bag (MWCO: 100 kDa) and incubating it at 37 °C in PBS (pH 7.4). At predetermined time intervals, the solution external to the dialysis bag was sampled, and the amount of DIP released was measured using the same approach as that for determining the drug-LE. Furthermore, to confirm the stability and controllability of the drug-release properties, DIP-FU-PPy NPs (0.6 mg/mL) were exposed to NIR irradiation (808 nm, 5 min, 2.0 W/cm^2^) at predestinated times after initiation of in vitro drug release; subsequently, the amount of DIP released was determined.

#### Analysis of photothermal characteristics

The photothermal performance was analyzed using a nanosuspension (0.6 mg/mL in PBS) in the aqueous phase exposed to an NIR laser (2.0 W/cm^2^, 808 nm, with an irradiation area of about 6 mm in diameter, PSU-III-LED, Changchun New Industries Optoelectronics Tech., China). The temperature distribution and quantitative temperature variation of the test aqueous solutions were verified with an IR thermal camera (A-BF, RX300, China) and thermocouple (TM-925, Lutron, Taiwan), respectively.

The photothermal conversion efficiency was calculated as previously described [33]. Briefly, the photothermal conversion efficiency is defined as the ratio of the increase in the internal energy of the aqueous solution to the total incident light (NIR) radiation [33]. The efficiency of photothermal conversion was calculated as [(aqueous solution mass (g) × aqueous fluid heat-capacity (4.2 J/(g/°C)) × average temperature increase (°C)]/[irradiated time (s) × (power density of NIR (W/cm^2^) × irradiated zone (cm^2^)] × 100%.

#### Drabkin’s assay and fluorescent fibrin clot microscopic assay for evaluating in vitro clot lysis

Blood plasma sampling from Wistar rats was approved by the Animal Ethics Committee of Taipei Medical University. Blood samples were collected from male Wistar rats (ranging ca. 250 to 300 g in body weight (BW)) into different tubes with 3.2% (w/v) sodium citrate (BD Vacutainer® Citrate Tubes – 363,083, USA). Centrifugation was performed for 10 min at 600 ×*g* (PURISPIN 17R, CRYSTE, UK) to obtain platelet-poor plasma (PPP). Platelet-rich plasma (PRP) from rat blood was obtained *via* centrifugation for 15 min at 200 ×*g* (PURISPIN 17R, CRYSTE). The tube was gently agitated to mix the PPP with PRP (PPP-PRP) in equal volumes. Platelets were then activated by supplementing PPP-PRP with thrombin receptor activator peptide (TRAP)-6. To evaluate thrombolysis in vitro, a colorimetric assay was performed to measure the release of red blood cells (RBCs) from blood clots using Drabkin’s approach [23]. Drabkin’s solution was composed of 38 mM KCN, 50 mM K_2_HPO_4_, 30 mM K_3_Fe(CN)_6_, and 2.5% w/v surfactant. Blood clots were created in vitro by blending RBCs and PPP-PRP with thrombin and CaCl_2_. The prepared blood clots were treated with different formulations (low dose (LD, 0.1 mg/mL), moderate dose (MD, 0.3 mg/mL), and high dose (HD, 0.5 mg/mL) of DIP-FU-PPy NPs) with or without NIR irradiation. After treatment, the supernatant was blended with an equal volume of Drabkin’s solution. The mixture was incubated at room temperature (*~* 22 °C) for 20 min, and the absorbance was measured at 540 nm using a microplate reader (EPOCH2, BioTek).

Artificial fibrin clots were made to assess in vitro fibrinolysis. Cyanine-5-n-hydroxysuccinimide (Cy5-NHS) ester at 0.3 mg/mL was used to conjugate fibrinogen. Artificial fibrin clots were fabricated by dissolving Cy5-congugated fibrinogen (2.5 mg/mL), thrombin (1 U), and CaCl_2_ (2.5 mM) in DI water, and single clots were placed in microtubes (1.5 mL) for 1 h at 37 ºC. Next, different samples with the same volume of fibrin clots were supplemented and co-incubated for another 1 h. Finally, the mixture was exposed to laser irradiation (808 nm) for 5 min. Fluorescent images were obtained *via* a fluorescence microscope (DFC7000T, Leica, Germany).

#### In vitro clot formation inhibition test

Fibrin clot generation was continuously monitored by determining the absorbance of the mixture at 340 nm for 1 h using a microplate reader (EPOCH2, BioTek). DIP (LD (0.01 mg/mL), MD (0.03 mg/mL), and HD (0.05 mg/mL)) and Fu (LD (0.03 mg/mL), MD (0.09 mg/mL), and HD (0.15 mg/mL)) were added to TRAP-6-pretreated PPP-PRP before the formation of fibrin clots to examine their effects on the inhibition of in vitro clot formation.

#### Spectrophotometric assessment of platelet aggregation inhibition

For platelet aggregation, TRAP-6-pretreated PPP-PRP (190 µL) was preincubated with 2 µL of a CaCl_2_ solution (10 mM) and 2 µL of test sample compounds (MD DIP or MD FU) at 37 °C for 2 min with orbital agitation (1000 rpm). Subsequently, a thrombin solution (final concentration, 0.4 IU/mL) was added, followed by further incubation for 10 min. The absorbance was measured at 595 nm using a suspension buffer as the negative control or blank, which was performed in triplicate. The PBS solution was used as a control group and was noted as 0% aggregation. Inhibition %: (1-Absorbance of test sample/Absorbance of blank) × 100% [34].

#### Platelet interactions with the formulations

Platelets of PPP-PRP were plated on cell plates with a glass coverslip. After attachment, platelets were stimulated with TRAP-6 (20 µg/mL) for activation. Non-activated (without TRAP-6 treatment) and activated platelets were incubated with different formulations (MD DIP or MD DIP-FU-PPy NPs), fixed with 4.0% formaldehyde, washed with PBS, and imaged using a fluorescence microscope (DFC7000T, Leica). Immunohistochemical (IHC) staining was used to assess the expression of P-selectin (NB100-65392, 1:500, 1 h, NOVUS) by cells, and observed by fluorescence microscopy (DFC7000T, Leica). Fluorescence intensities were quantified using ImageJ software.

#### In vitro BAEC studies

In total, 10^5^ BAECs were cultured in a confocal laser scanning microscope (CLSM) dish until confluency. To mimic and induce inflammation, lipopolysaccharide (LPS, 0.1 µg/mL) was added, and cells were incubated at 37 °C in a humidified incubator overnight. IHC staining was used to assess P-selectin expression (NB100-65392, NOVUS, 1:500, 1 h) in cells, and nuclei were counterstained with DAPI (1:1000, 30 min) or PI (1.5 µM, 30 min) and observed by fluorescence microscopy (DFC7000T, Leica). Fluorescence intensities were quantified using ImageJ software. Endogenous inflammation was measured by staining with the fluorescent reactive oxygen species (ROS) biomarker, 2’,7’-dichlorodihydrofluorescein diacetate (DCFH-DA, Sigma-Aldrich, 1 mM), for 30 min at 37 °C. An untreated group served as the control. To quantify cellular interactions with the test nano-formulations, cells (with or without LPS) were treated with DIP-FU-PPy NPs (with particle sizes of < 300 nm, 0.6 mg/mL) for 40 min. After treatment, cells were washed twice with warm PBS (pH 7.4). Fluorescent signals were acquired to elucidate differences in the extents of ROS, P-selectin, and DIP-FU-PPy NPs using fluorescence microscopy (DFC7000T, Leica). Fluorescence intensities were quantified using ImageJ software.

### In vivo animal model studies

The in vivo animal studies (using 60 male ICR mice at ca. 25 g in BW and nine male Wistar rats at ca. 300 g in BW) obtained from BIOLASCO (Taipei, Taiwan) were approved by the Taipei Medical University Animal Ethics Committee (LAC2022-0208 and LAC-2019-0023). Surgery was performed under inhalant isoflurane anesthesia (2–4%). To create a mesenteric thrombus ICR rodent model, anesthetized animals were placed on a Petri dish, the intestinal organs were mildly exteriorized, and the mesenteric tissues were gently spread out to identify vessels. Filter paper soaked in a 35% FeCl_3_-6H_2_O solution was positioned on the identified vessel. The formulations (DIP and DIP-FU-PPy NPs) were systemically and individually given to thrombotic ICR mice (MD DIP equivalent: 3 mg/kg BW, 0.1 mL per mouse). To perform in vivo NIR-photothermal imaging, the vessels of anesthetized animals were irradiated with an 808-nm laser at a power density of 2.0 W/cm^2^ for 10 min. A thermographic camera (A-BF, RX300, China) was used to obtain detailed thermal images of vessels in vivo. After administration and NIR exposure, animals were sacrificed using CO_2_. In addition, fluorescence imaging was performed on the treated vessels using an in vivo imaging system (IVIS, IVIS Lumina III XRMS, PerkinElmer, USA). Test ICR mice were sacrificed after NIR treatment, and the vessel tissues were removed.

#### Evaluation of in vivo thrombolysis and biodistribution

In the vascular penetration study, the MD of DIP-FU-PPy NPs or different doses of DIP (MD (3 mg/kg BW) or HD (5 mg/kg BW)) in PBS were slowly administered by a systemic injection. Fluorescence microscopy (DFC7000T, Leica) was used to assess the DIP signal representing vessel accumulation and understand thrombus targeting and the penetration ability 10 min after systemic administration of the formulations. All harvested vessels were fixed in 10% formalin, embedded in paraffin blocks, and cut into approximately 8-µm sections. The mean fluorescence intensity of DIP in mouse thrombi was analyzed using ImageJ software. In the metabolism biodistribution study, 0.3 mg/mL Cy5-NHS ester was first used to conjugate the amine of the Py monomer (10 mg), and then DIP-FU-PPy NPs were used following the same protocol as mentioned above. DIP or Cy5-conjugated DIP-FU-PPy NPs were given to test mice. At 1, 3, 5, and 7 days post-administration, the soft organs (heart, liver, spleen, lungs, and kidneys) of treated animals were harvested, and the PPy nanocarrier signal (Cy5) and DIP fluorescent signals of tissues were determined using IVIS (IVIS, IVIS Lumina III XRMS).

Antithrombotic activity was evaluated using a detailed in vivo molecular mechanism after treatment. In the efficacy study, ICR mice were randomly divided into the following groups:1) healthy untreated group; 2) embolized thrombosis + NIR laser group; 3) embolized thrombosis + DIP + NIR laser group; 4) embolized thrombosis + DIP-FU-PPy NPs + NIR laser group; 5) embolized thrombosis + mixture of DIP plus PPy (without FU) + NIR laser group; 6) embolized thrombosis + DIP-FU-PPy NPs group; 7) embolized thrombosis + FU-PPy NPs + NIR laser group; and 8) embolized thrombosis + DIP-FU-PPy NPs + NIR laser with 1 day preservation group (*n* = 6). Drug-treated mice received an equivalent dose of DIP (3 mg/kg BW). As previously described, a ferric chloride-induced thrombosis ICR mouse model was established using this method. Mouse vessels were exposed to a laser (808 nm, 2.0 W/cm^2^) 10 min post-administration for 10 min. Subsequently, test animals were sacrificed using carbon dioxide, and the vessels were harvested, fixed in 10% formalin, dehydrated using an alcohol gradient, embedded in paraffin wax, and sectioned for histological measurement of the thrombolytic efficacy.

Histological sections were further stained with hematoxylin and eosin (H&E) (Sigma, St. Louis, MO, USA) to assess the histological morphology of the treated vessels. An anti-P-selectin antibody (NB100-65392, 1:100, 1 h), anti-heat shock protein antibody (HSP 1:100, 1 h), dichlorodihydrofluorescein diacetate (DCFH-DA, 40 µM, 30 min), and anti-plasminogen activator inhibitor (PAI)-1 antibody (1:100, 1 h) were also used for immunochemical staining and microscopic observation. The clot-removal efficacy was analyzed, using a histological method, as vascular clot area/lumen area) × 100%. Calculated areas and microscopic fluorescence intensities were ascertained using ImageJ software. In the in vivo ultrasonic imaging study, a Doppler ultrasound flow detector (Sqarq, Philips, The Netherlands) was applied to the vessel to measure the vascular flow velocity.

#### In vivo biosafety and bleeding assays

The major soft organs of treated animals, including the spleen, lungs, heart, kidneys, and liver, were harvested after NIR treatment. The organs were fixed in 10% formalin. Fixed tissues were subsequently embedded in paraffin blocks and cut into sections approximately 8 μm thick. Histological sections were stained with H&E and examined under a microscope (DFC7000T, Leica). Blood serum hematological tests (RBCs), hemoglobin (HGB), hematocrit (HCT), mean corpuscular volume (MCV), and white blood cells (WBCs)) were performed using a hematology analyzer (Procyte Dx, IDEXX Laboratories, USA).

For the bleeding test, the about distal 1-cm section of a mouse tail was cut off using a scalpel and submerged in PBS pre-warmed to 37 °C after administration and treatment with DIP-FU-PPy NPs or a similar amount of DIP. Animals were observed for 30 min to determine the extent of bleeding. Further, blood cells were centrifuged for 10 min at 4000 *g*. Cell pellets were resuspended in 1% Triton X-100 (1 mL), and the bleeding volume/extent was assessed based on the hemoglobin level in the PBS solution by determining the absorbance spectra of hemoglobin (*n* = 6).

The hemostatic ability was also assessed using a mouse hepatic hemorrhage model [35]. To perform the experiment, mice were first anesthetized. Pre-weighed filter paper was carefully positioned under the liver, and bleeding was induced using a 20-gauge needle. The extent of bleeding was assessed, and the total amount of blood loss was quantified by weighing the filter paper.

### Statistical analysis

Experimental results are presented as the mean plus standard deviation (SD). Differences between groups were statistically analyzed for significance using Student’s *t*-test or Kruskal-Wallis test. Differences were considered statistically significant at *p* < 0.05.

## Results and discussion

Herein, we studied the characteristics of the prepared nano-formulation (DIP-FU-PPy NPs). We encapsulated an antithrombotic agent, DIP, into this nanocarrier system and evaluated its material properties, such as drug-LE, particle size, zeta potential, morphological properties, NIR-photothermal properties, in vitro cellular interactions, drug-release behavior, and physiochemical properties. We also evaluated its functions, such as inhibition of clot formation, antiplatelet aggregation, and clot lysis in vitro, effect of P-selectin-mediated cell accumulation of DIP-FU-PPy NPs, and the combined therapeutic effect of DIP-FU-PPy NPs. Ferric chloride was used to induce fibrin-insoluble mesenteric thrombi in an in vivo ICR mouse model. When reaching the target thrombus site after systemic administration, the combined therapeutic effect of non-invasive NIR nano-photoablation and the release of therapeutic DIP was expected to act against the thrombus, resulting in targeted loosening with resolution of the formed clot. We hypothesized that the targeted therapeutic mechanism of DIP-FU-PPy NPs involved precise drug delivery with phototherapeutic behaviors, thrombus-targeting and penetration performance, and safe and efficient thrombus management, which were validated using biochemical, histological, and in vivo examinations.

### Material preparation and physicochemical characterization

**Fig. 1:**
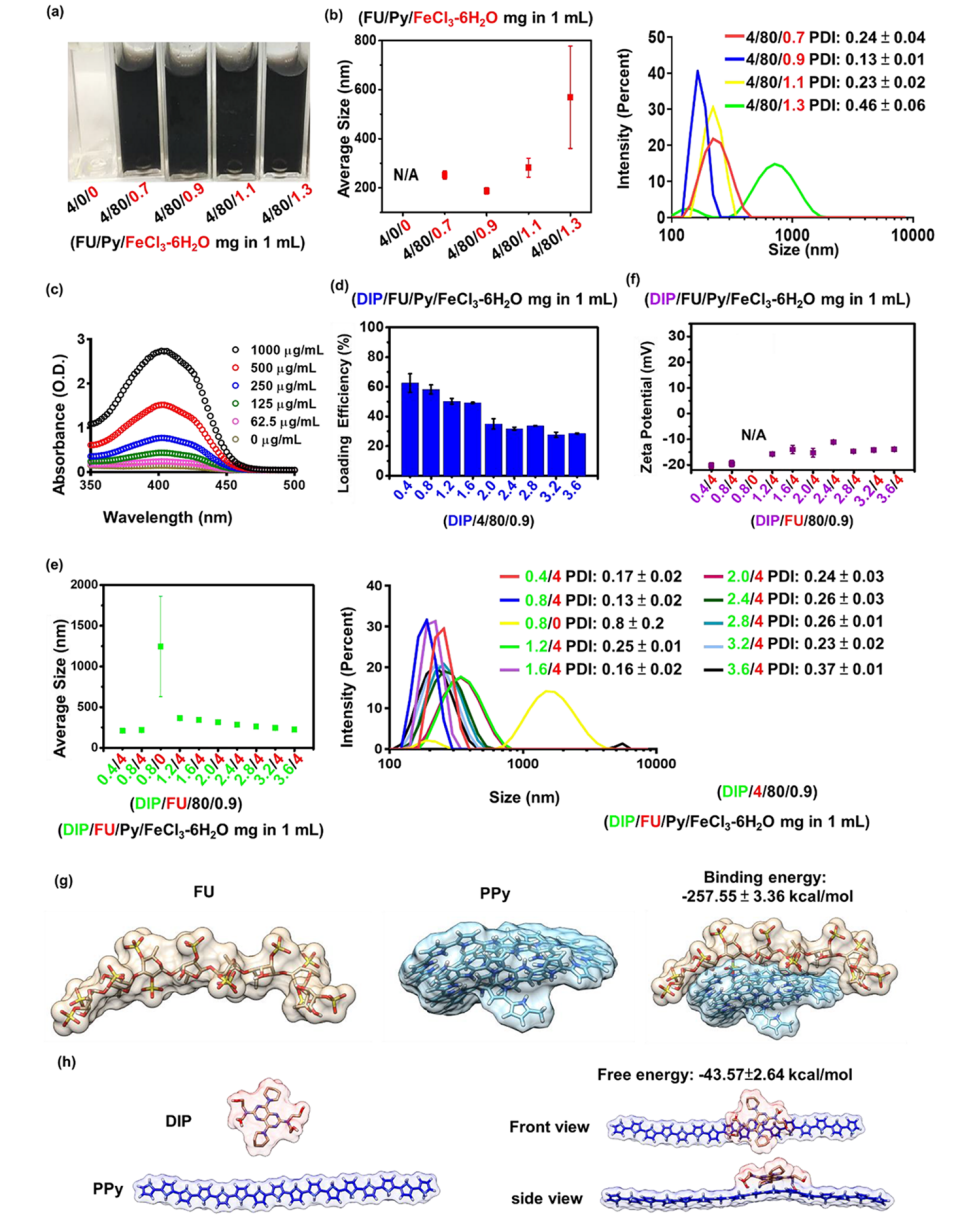
(**a**) Photographic observations showing that different ratios of pyrrole (Py)/ferric chloride (FeCl_3_-6H_2_O) reacted with fucoidan (FU) to optimize the formulation (FU/Py/FeCl_3_-6H_2_O 4/80/0.9 mg per mL). (**b**) Size distributions of FU-polypyrrole (PPy) formulations (including FU/Py/FeCl_3_-6H_2_O 4/80/0.9 mg per mL) as measured by dynamic light scattering (DLS, Malvern Zetasizer, Nano-ZS, ZEN 3600). (**c**) Spectrophotometric data measured by an UV-Vis spectrophotometer (EPOCH2, BioTek) revealing optical properties of dipyridamole (DIP), whose absorbance intensity increased in a dose-dependent manner, ranging from 350 to 460 nm. (**d**) Drug-loading efficiency (LE) and DLS data showing (**e**) the size distribution and (**f**) zeta potential of different formulations (under a fixed component of FU/Py/FeCl_3_-6H_2_O at 4/80/0.9 (mg) per mL). DIP data showed that the loading efficiency of DIP decreased with an increase in the DIP dosage because the polymeric payload lacked a sufficient capacity to encapsulate the excess DIP. (**g and h**) Molecular dynamic simulation data showing the binding energy between FU and PPy or DIP and PPy

A small size (10–300 nm) of hydrophilic NPs is a common prerequisite for the successful delivery for biomedical use [36]. Hydrophobic PPy can be synthesized *via* aqueous chemical oxidative polymerization in the presence of ferric ions. During PPy polymerization, blending FU with an anionic amphiphilic structure stabilizes the PPy. As shown in Fig. 1a, under the same feed concentrations of 4 mg/mL FU, photographic observation of the solution appeared nearly transparent before supplementation with the FeCl_3_-6H_2_O group. No particle formation was observed without supplementation of the FeCl_3_-6H_2_O group, whereas particles were formed with increasing average particle sizes (from approximately 187 to 569 nm) when the FeCl_3_-6H_2_O amount increased (from 0.9 to 1.3 mg/mL) (Fig. 1b). According to a previous study, PPy formation increases with increasing concentrations of ferric chloride [37]. FU could not stabilize the excess PPy in the 1.3 mg/mL FeCl_3_-6H_2_O group, resulting in hydrophobic interactions of PPy-driven aggregation (approximately 569 nm) in the aqueous phase. When the amounts of FeCl_3_-6H_2_O were relatively lower (at 0.7, 0.9, and 1.1 mg/mL), average particle sizes were < 300 nm (Fig. 1b). To obtain the smallest nanocarrier, a given amount of FeCl_3_-6H_2_O (0.9 mg/mL) was selected for further drug encapsulation.

In the drug-encapsulation study, the added hydrophobic DIP was encapsulated during the polymerization of PPy through hydrophobic interactions owing to its hydrophobicity. Anionic FU may be able to form nano-shells around the formed DIP-PPy. During nano-formation, the core (DIP-PPy)-shell (FU) nanocomplex (DIP-FU-PPy NPs) was generated by self-assembly in the aqueous phase. The DIP-LE, as determined by spectrophotometry, showed that the DIP absorbance intensity increased in a dose-dependent manner, ranging from 350 to 460 nm (Fig. 1c). The aromatic structure and π-system of DIP were responsible for the high fluorescence and absorption [38].

The DIP drug-LE decreased with an increase in the DIP dosage because the polymeric payload lacked sufficient capacity to encapsulate the excess DIP (Fig. 1d) [39]. The DIP/FU/Py/FeCl_3_-6H_2_O test groups (0.4/4/80/0.9 and 0.8/4/80/0.9 (mg) per mL solution) showed acceptable LEs [40] (DIP LEs of ca. 60%). Consistent with earlier findings, PPy showed a suitable drug-loading capacity [41]. The 0.8/4/80/0.9 DIP/FU/Py/FeCl_3_-6H_2_O group exhibited slightly larger particle sizes (~ 220 nm, Fig. 1e) compared to those of 0.4/4/80/0.9 DIP/FU/Py/FeCl_3_-6H_2_O at ~210 nm, possibly because of the presence of more DIP existing in single NPs in the 0.8/4/80/0.9 DIP/FU/Py/FeCl_3_-6H_2_O group. In the 1.6/4/80/0.9 DIP/FU/Py/FeCl_3_-6H_2_O group, its LE was low (49.2%), and particle sizes were rather large (344 nm), compared to the 0.8/4/80/0.9 DIP/FU/Py/FeCl_3_-6H_2_O group. Thus, 0.8/4/80/0.9 DIP/FU/Py/FeCl_3_-6H_2_O was used for subsequent experiments, for which the calculated drug-LC was ~10%. The drug-LC reflects the mass ratio of drugs to nanomedicines. Considering the recovery result, it was noted that not all of the Py had been polymerized in this carrier system during the synthesis process.

The particle size was reduced from the range of 1.2/4/80/0.9 to 3.6/4/80/0.9 particles (Fig. 1e). Zeta potentials of the prepared nano-formulations slightly increased with an increase in the DIP feed amount (Fig. 1f) owing to contributions from the faintly cationic DIP [42]. Compared to when FU was present (in the 0.8/4/80/0.9 DIP/FU/Py/FeCl_3_-6H_2_O group, approximately 220 nm and − 20 mV), the group lacking FU (0.8/0/80/0.9 DIP/FU/Py/FeCl_3_-6H_2_O) showed an increased particle size to the micro-size range (above 1000 nm) and no available zeta potential (N/A) (Fig. 1e, f). In some experimental groups (4/80/1.3 FU/Py/FeCl_3_-6H_2_O and 0.8/0/80/0.9 DIP/FU/Py/FeCl_3_-6H_2_O), their large extents of standard deviation of the average particle size and higher PDI values (*>* 0.3, as per the left side distribution data) indicated colloidal instability during formulation. These findings confirmed that excess DIP or a lack of Fu stabilization in the carrier system resulted in an aggregation or precipitation phenomenon.

This indicates that FU, as an anionic and amphiphilic surfactant, allows cationic PPy and DIP to quickly co-assemble into stable NPs driven by hydrophobic ionic forces. This prevented aggregation and enhanced the dispersive stability of NPs by increasing the surface charge and electrostatic repulsion or by reducing the interfacial energy between the solvent and particles. The molecular dynamic simulation suggested that the binding energy (-257.55 ± 3.36 kcal/mol) between Fu and PPy and the binding energy (-43.57 ± 2.64 kcal/mol) between DIP and PPy could be the reason behind the self-assembly mechanism (Fig. 1g, h). The molecular level analysis further suggested that NPs functionalized with PPy plus Fu were structurally stable. The molecular dynamic simulations and calculated Fu-PPy binding free energy confirmed the experimental results, showing better binding of the drug to the copolymer.

#### Nano-formation, morphological change, and photothermal responsiveness assessments

**Fig. 2:**
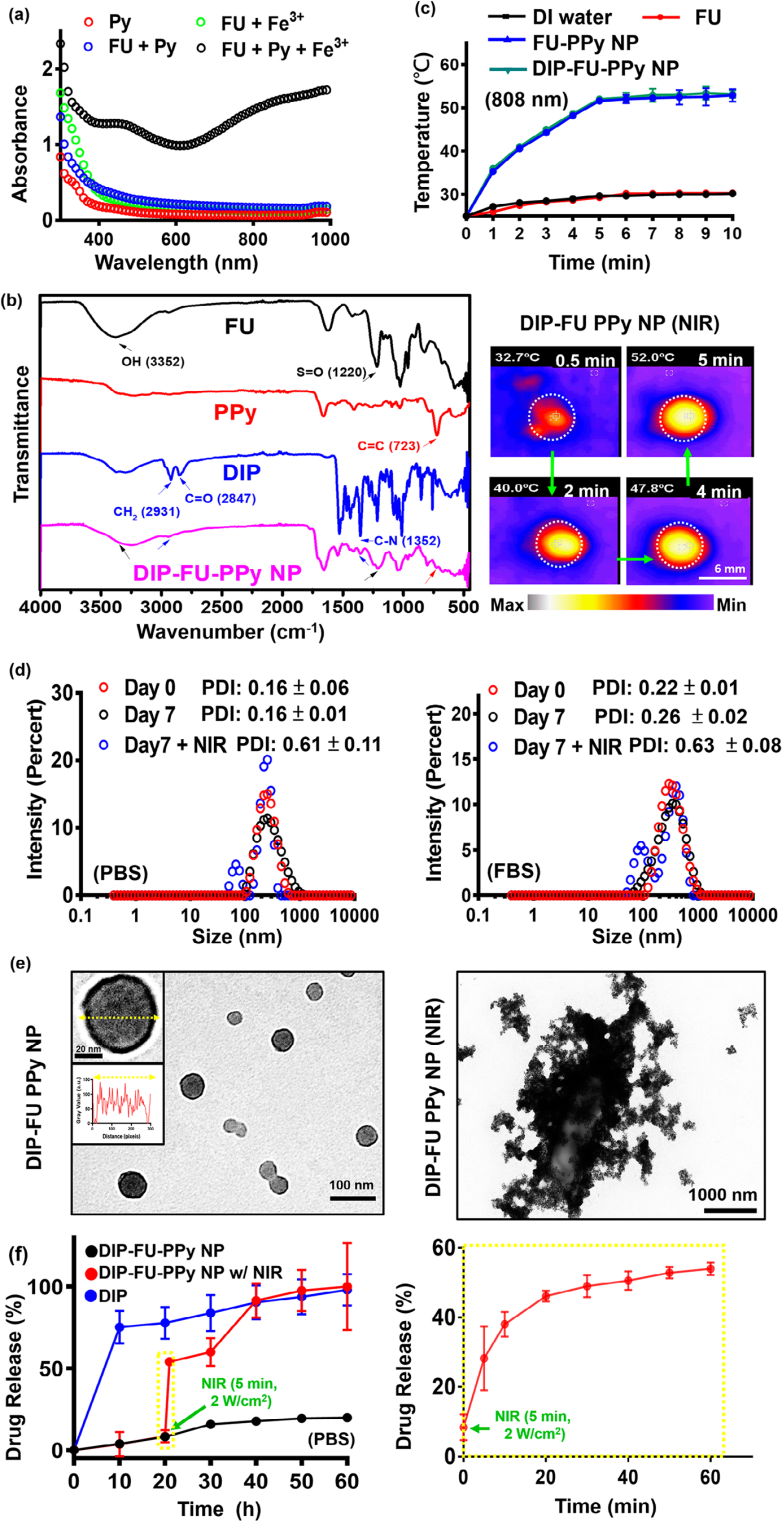
(**a**) UV-Vis (300 to 1000 nm) absorption spectroscopic analysis of the formation of polypyrrole (PPy) using ferric chloride. (**b**) Fourier transform infrared (FTIR) results showing the chemical structure of the prepared samples. (**c**) Results of temperature changes of the test liquid formulation after NIR treatment showing quantitative analysis with a thermocouple (TM-925, Lutron) until the temperature reached 25 °C, and a qualitative image of these aqueous formulations with a thermal camera (A-BF, RX300, China) at 808 nm and 2.0 W/cm^2^ (scale bar: 6 mm). (**d**) Polydispersity index (PDI) and (**e**) TEM (scale bar: 100 nm) results showing morphological changes of the spherical core-shell structure of the nano-formulation with colloidal stability (PBS or PBS plus 10% FBS at pH 7.4 for 7 days). After laser irradiation, the TEM image shows particle disruption and aggregation (scale bar: 1000 nm). (**f**) Spectrophotometric analysis showing the release of dipyridamole (DIP) from the nanoparticles (NPs) under PBS. Under irradiation with NIR light, the photothermal effect of the DIP-fucoidan (FU)-PPy NPs triggered the morphological transformation of the polymeric carrier, which allowed the DIP to be released from the NPs

A UV-Vis spectroscopic method [43] was used to confirm the mechanism of PPy formation in the FU-PPy NP system. Figure 2a displays the UV-Vis spectroscopic data of the Py monomer (0.2 mg/mL, red), solution (green) of FU (0.2 mg/mL) with ferric chloride (0.2 mg/mL), and solution (blue) of Py monomer (0.2 mg/mL) with FU (0.2 mg/mL). The UV-Vis spectroscopic data showed no obvious absorption at 600 − 800 nm (FU + ferric chloride, and FU + Py group) in the spectrum, suggesting the absence of Py monomer polymerization [43]. Aqueous solutions (black) containing the Py monomer (0.2 mg/mL) and FU (0.2 mg/mL) were mixed with ferric chloride (0.2 mg/mL), and we found a stronger intensity at 600 − 800 nm. This could be characteristic of the π − π* transition of PPy materials with higher molecular weights (polymerization of Py monomers) [43]. These findings confirmed that ferric chloride was involved in the oxidative polymerization of the Py monomer in this FU-PPy NP system. Similar findings support this oxidative method for Py polymerization using ferric chloride [31]. In the case of NPs, it might not be pure absorption, but extinction (absorption plus scattering), which is detected by UV-Vis spectroscopy. Fourier transform IR (FTIR, Fig. 2b) spectroscopy provided resolvable chemical structural information of test samples. The existing FU polymer, including its nano-construction, displayed characteristic peaks, representing a hydroxyl group (OH, ca. 3352 cm^− 1^) with an S = O group (ca. 1220 cm^− 1^). PPy revealed peaks at C = C (ca. 723 cm^− 1^). DIP revealed its structure at CH_2_ (ca. 2931 cm^− 1^) and at C-N (ca. 1352 cm^− 1^). The resultant DIP-FU-PPy NPs indicated similar positions as mentioned (C–N at ca. 1352 cm^− 1^, N-H at ca. 3391 cm^− 1^, CH_2_ at ca. 2931 cm^− 1^, S = O at ca. 1220 cm^− 1^, C = C at ca. 723 cm^− 1^, and an OH functional group near 3352 cm^− 1^). The FTIR and UV-Vis absorption spectroscopic analyses confirmed that DIP-FU-PPy NPs had been synthesized.

The photothermal effect of the tested nano-formulations after NIR irradiation showed that the PPy-based NPs produced hyperthermia in subsequent applications (Fig. 2c). The temperature of the DIP-FU-PPy NP or FU-PPy NP group increased upon NIR irradiation and reached a plateau after 5 min. The efficiency of photothermal conversion of DIP-FU-PPy NPs or FU-PPy NPs was ca. 25%, which was higher than that of FU or water alone. Gold-based NPs (AuNPs) have been investigated as photoablation agents for cancer treatment. However, NIR-absorbing AuNPs have limited photostability because their absorption peaks and structures may degrade after prolonged laser exposure [20]. Among NIR-absorbing nanomaterials, PPy-based materials are particularly important as photoablation substances for regional PTT. According to previously published literature, NIR-conducting polymeric-based nanomaterials showed a similar rate of elevated temperature (ca. 10 °C/min) that produced biocompatible mild hyperthermia for biomedical applications [31].

To evaluate the stability of the DIP-FU-PPy NPs in vitro, the NPs were incubated with a PBS solution and PBS containing 10% serum (10% FBS) for 7 days at 37 °C. The PDI of DIP-FU-PPy NPs remained close to 0.15 in PBS for 7 days, indicating that colloidal stability was maintained (Fig. 2d). The particle size and PDI of DIP-FU-PPy NPs slightly increased in these media, but the PDI was still less than 0.27 up to 7 days, indicating that no obvious aggregation had occurred (Fig. 2d). The TEM analysis revealed changes in the morphology and size of the NPs before and after NIR irradiation (Fig. 2e). The NPs were spherical with a core-shell structure prior to laser irradiation, and possessed an inner core PPy structure and an outer FU shell made of different components. After laser irradiation, the particle size and PDI (up to 0.6) significantly increased, as revealed by the TEM and PDI analyses (Fig. 2d, e).

#### In vitro drug-release studies

The drug-release behavior of DIP was investigated by dialysis, both with and without external stimuli. As shown in **Fig. 2f**, less than approximately 10% of DIP was released from the NPs in the dialysis buffer over a period of 10 h (black). DIP was released from DIP-FU-PPy NPs in a more-sustainable manner in a PBS solution, and that release was steady for up to 20 h (approximately 10% lower DIP was released), suggesting that DIP-FU-PPy NPs could retard the DIP release rate (Fig. 2f). The initial burst release of DIP was thus reduced after being encapsulated in FU-PPy NPs. The effect of retardation can be attributed to stable interactions between DIP and the FU-PPy NPs, which should be similar to electrostatic interactions between the anionic FU and cationic DIP. Furthermore, PPy, with its hydrophobic domains, can easily bind to DIP. Thus, the DIP-FU-PPy NP complex formed was responsible for the decreased DIP release rate. However, the cumulative release percentage of free DIP increased rapidly to ca. 70% after 10 h (blue).

The hyperthermic temperature generated by the embedded conductive PPy under NIR light triggered the release of DIP (Fig. 2f, red). The FTIR analysis revealed that no structural changes occurred between the hyperthermia-treated DIP group and the no hyperthermia-treated DIP group, indicating the chemical stability of DIP (Fig. 1S). After initial NIR irradiation, the cumulative release percentage of DIP sharply increased by ca. 4-fold compared to that in the non-NIR-treated DIP-FU-PPy NP group, representing a notably accelerated DIP release process (Fig. 2f). Upon irradiation with NIR light, the NIR-photothermal effect of DIP-FU-PPy NPs triggered a morphological transformation of the polymeric carrier and increased the PDI value to 0.6, as per DLS and TEM data (Fig. 2d, e). This allowed the therapeutic DIP to be released from the NPs and affect the thrombotic vessel. These data indicated that the constructed DIP-FU-PPy NPs with a core-shell structure could avoid random leakage of the laden DIP into the blood circulation but then hasten drug release at the lesion site under NIR stimuli, thus generating maximum therapeutic efficacy and minimal side effects. Despite the promising applications of this developed drug nanocarrier, its metabolism and potential toxicity remain to be fully elucidated, and further investigations are needed.

#### In vitro clot-lysis activity

**Fig. 3:**
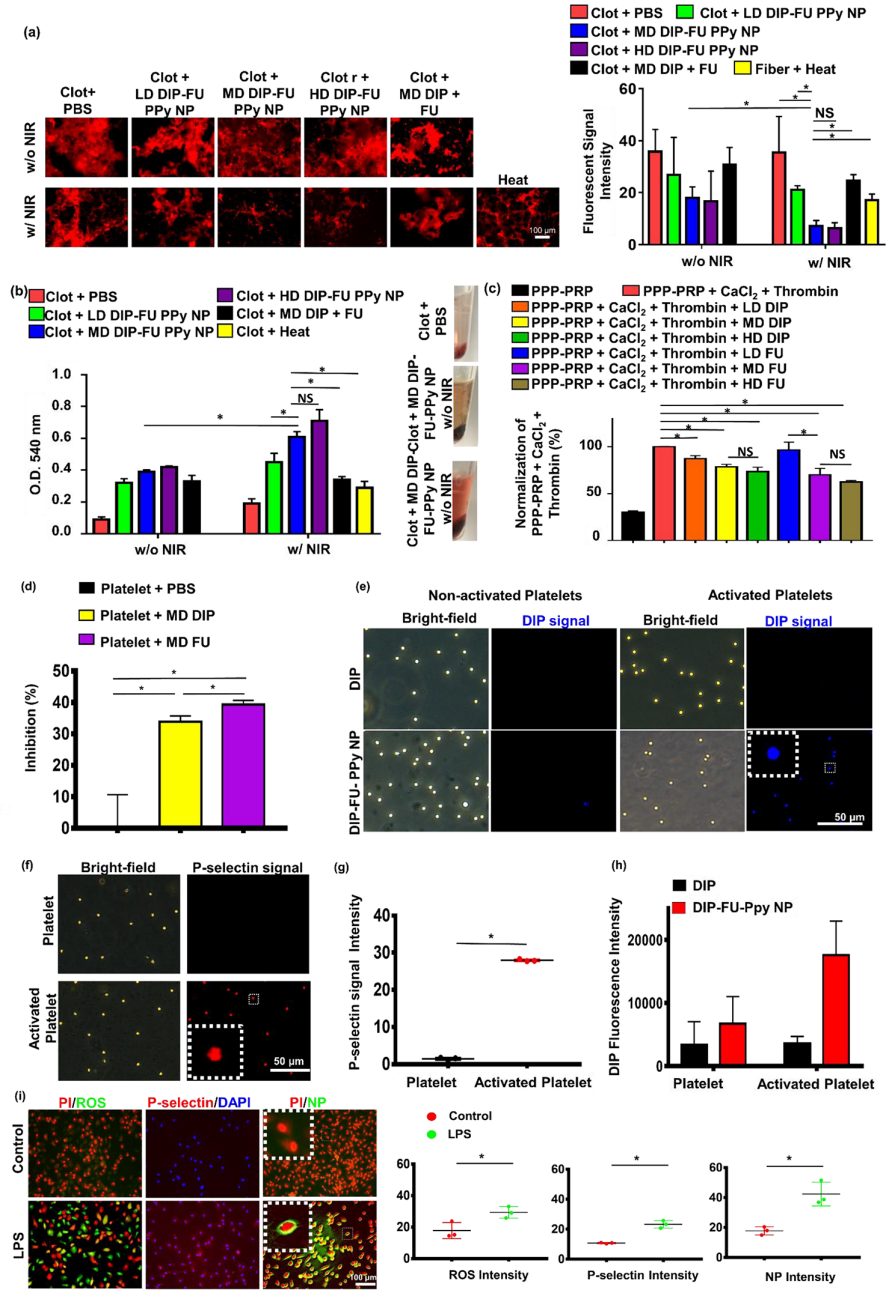
Thrombolysis and in vitro clot-formation assay. Thrombolytic effect: supplementation with a low dose (LD), moderate dose (MD), and high dose (HD) of dipyridamole (DIP)-fucoidan (FU)-polypyrrole (PPy) nanoparticles (NPs) to in vitro clots. The quantitative thrombolytic activity of formulations was evaluated using (**a**) a microscopic assay (DFC7000T, LEICA) and (**b**) a colorimetric assay to assess the release of red blood cells (RBCs) from blood clots via Drabkin’s method. (**c**) Inhibition of in vitro clot formation using colorimetric assays: the addition of LD, MD, and HD of DIP or FU to platelet-poor plasma (PPP)-platelet-rich plasma (PRP) (inhibition of in vitro clot formation) before CaCl_2_ was added (ECHO CHEMICAL, 2.5 mM) and thrombin (Sigma-Aldrich, 1 U/mL). (**d**) Platelet aggregation using colorimetric assays: the addition of the MD of DIP or FU to PPP-PRP in the presence of CaCl_2_ and thrombin. (**e**) Fluorescence microscopic (DFC7000T, LEICA) images of non-activated and activated platelets (induced by TRAP-6, 10 µM) treated with MD DIP and DIP-FU-PPy NPs (scale bar: 50 μm). (f) Cellular P-selectin levels assessed by microscope. Quantitative (**g**) P-selectin and (**h**) DIP levels of non-activated platelets or activated platelets using a microscopic assay. (**i**) Fluorescent microscopic data showing a fluorescence image of the nano-formulation interacting with or without LPS-induced bovine aortic endothelial cells treated with 4’,6-giamidino-2-phenylindole (DAPI, Biotium #40,043, 1:1000, 30 min, 23 °C), propidium iodide (PI, ThermoFisher, 1.5 µM, 30 min, 23 °C), or 2ʹ,7ʹ-dichlorofluorescein diacetate (DCFH-DA, Sigma-Aldrich, 40 µM, 30 min, 37 °C) for ROS, and NB100-65392 (1:500, 1 h, 37 °C) as a P-selectin marker (scale bar: 100 μm). Control group: cells without LPS treatment. (* *p* < 0.05; NS, non-significant at *p* > 0.05)

To verify the bioactivity of the DIP-FU-PPy NPs at different doses (LD, MD, and HD), an in vitro blood clot*-*lysis assessment was performed using fluorescent fibrin clot microscopic and Drabkin’s [23] methods (Fig. 3a, b). Without 808-nm NIR (Fig. 3a), the LD, MD, and HD of DIP-FU-PPy NPs (0.1, 0.3 and 0.5 mg/mL) of the fluorescent fibrin clot were respectively approximately 1.34-, 1.98-, and 2.15-fold lower than those of the PBS group, indicating that the FU-decorated nano-formulation partially aided in vitro clot lysis. Upon irradiation with 808-nm NIR (Fig. 3a), clot absorbances of the LD, MD, and HD of DIP-FU-PPy NPs were respectively approximately 1.68-, 4.99-, and 5.54-fold lower than those of the PBS group, indicating that NIR irradiation-driven hyperthermia with NIR-triggered DIP release further facilitated in vitro clot lysis. These fluorescent data suggested that NIR-driven hyperthermia strengthened the ability of the DIP-FU-PPy NPs to perform in vitro thrombolysis. The thrombolytic activity of MD DIP-FU-PPy NPs was significantly superior to that of the LD. Nevertheless, no significant differences were found between the MD and HD of DIP-FU-PPy NPs (Fig. 3**a**). The thrombolytic performance was further examined using Drabkin’s method to colorimetrically assess the release of RBCs from the prepared clots (Fig. 3b). Generally, when the extent of thrombolysis increases, more RBCs are released from the clot, thereby increasing the absorbance of the supernatant. Here, the absorbance of the red supernatant at 540 nm intensified as the extent of thrombolysis increased; this increase was proportional to the color change.

Without 808-nm NIR irradiation (Fig. 3b), the clot absorbances given for the LD, MD, and HD of DIP-FU-PPy NPs (0.1, 0.3, and 0.5 mg/mL) were approximately 3.56-, 4.33-, and 4.67-fold greater, respectively, than those of the PBS group, indicating that FU decoration of the nano-formulation partially aided in vitro clot lysis. The clot + PBS control group showed a lower absorbance at 540 nm, indicating fewer released RBCs. According to previously published findings, FU possesses antiatherosclerotic and antithrombotic effects [44, 45], which support our results. Upon irradiation with 808-nm NIR (Fig. 3b), the clot absorbances of the LD, MD, and HD of DIP-FU-PPy NPs were approximately 2.37-, 3.21-, and 3.74-fold greater, respectively, than those of the PBS group, indicating that NIR irradiation-driven hyperthermia with NIR-triggered DIP release further facilitated in vitro clot lysis. The clot + PBS plus NIR control group showed a lower absorbance at 540 nm, indicating that fewer RBCs had been released. These findings proved that localized NIR-driven hyperthermia augmented thrombolysis by DIP-FU-PPy NPs in vitro. The thrombolytic activity of MD DIP-FU-PPy NPs was significantly higher than that of the LD. However, no significant differences were observed between the MD and HD of DIP-FU-PPy NPs (Fig. 3b). Therefore, the MD simulation of DIP-FU-PPy NPs was selected for further experiments.

Several studies demonstrated that DIP and FU exhibit antiplatelet properties and inhibit clot formation [46, 47]. In turbidimetric assays, we verified the generation of fibrin clots by supplementing PPP-PRP with CaCl_2_ and thrombin. Next, FU or DIP at various doses was supplemented with PPP-PRP together with agonists and CaCl_2_ to examine the corresponding effects on inhibiting clot formation. As shown in Fig. 3c, FU and DIP decreased the rate of fibrin-clot formation, according to the fibrin clot absorbance at 340 nm [48]. The lower absorbance of the FU- and DIP-containing samples in a dose-dependent manner signifies that FU and DIP had inhibitory effects against the formation of fibrin clots. To inhibit platelet aggregation, FU or DIP was added to PPP-PRP together with agonists and CaCl_2_ to examine the corresponding effects on the inhibition of platelet aggregation.

Platelet aggregation is a method employed to evaluate platelet activation levels and the extent of platelet-platelet binding in response to agonists [49]. Spectrophotometric assessment is a common technique used to determine the aggregation activity of platelets in vitro [50]. As shown in Fig. 3d, FU and DIP increased the inhibition rate of platelet aggregation according to the platelet aggregation absorbance at 595 nm [51]. A higher (platelet aggregation) percent inhibition of the FU- and DIP-containing samples than that of the PBS group was observed, indicating that FU and DIP had inhibitory effects against platelet aggregation. These results are consistent with studies reporting that inhibition of activated platelet aggregation and clot formation are bioactivities of FU and DIP [46, 47]. DIP was demonstrated to exert its antithrombotic effects through various mechanisms. These include inhibiting platelet activation, affecting platelet function, inhibiting the reuptake of adenosine by erythrocytes (which can lead to vasodilation), influencing the expression of endothelial activation markers such as von Willebrand factor antigen, and potentially having anticoagulant effects by reducing peak and total thrombin generation after a transient ischemic attack or ischemic stroke [52]. According to previously published findings, FU was shown to inhibit platelet activation through mechanisms such as reducing the concentration of endothelial microparticles and von Willebrand factor, as well as inhibiting the flow of extracellular calcium ions [53].

A significant increase in absorbance at 540 nm was observed after the in vitro clots were treated with the FU-decorated nano-formulation (MD DIP-FU-PPy NPs upon NIR irradiation; Fig. 3b), suggesting that the lytic performance significantly increased owing to the NIR-photothermal responsive FU-decorated DIP nanocarrier. FU has high binding affinity toward P-selectin. Therefore, an improvement in the therapeutic effect of DIP-FU-PPy NPs (Fig. 3) could also have been due to the increased accumulation of therapeutics in the thrombus by targeting P-selectin-overexpressing cells (activated platelets).

#### P-Selectin delivery and activated platelet interactions

Binding capacities of DIP-FU-PPy NPs to non-activated and activated platelets were assessed using fluorescence microscopy. Non-activated platelets treated with DIP-FU-PPy NPs displayed weak DIP fluorescence signals (blue). However, the DIP intensity considerably increased in activated platelets compared to that in non-activated platelets treated with DIP-FU-PPy NPs or activated platelets treated with DIP. The fluorescence intensity (verified using ImageJ software) of DIP-FU-PPy NPs in activated platelets was higher than those of DIP in non-activated and activated platelets, respectively (Fig. 3e-h). The fluorescence intensity indicated that FU affected the specific binding of DIP-FU-PPy NPs to activated platelets, further verifying the ability of Fu-decorated NPs to precisely deliver therapeutics to the thrombus site. Fluorescence microscopic data confirmed that the P-selectin level in activated platelets had increased 20-fold compared to that in non-activated platelets (Fig. 3f, g).

In addition to activated platelets, LPS-treated endothelial cells exhibit inflammation similar to that seen during the progression of thrombosis [54] and induce intracellular P-selectin [55]. As shown in Fig. 3i, LPS-treated cells indeed yielded a significant increase in intracellular inflammatory markers (ROS, approximately 1.65-fold stronger than in the control group) and P-selectin (approximately 2.17-fold stronger than in the control group), suggesting that inflamed endothelial cells had developed in vitro [56]. DIP [57] can inhibit blood clot formation when administered at higher doses over a short period owing to its hydrophobicity. Conventional drug delivery cannot cause its specific accumulation in thrombotic regions, possibly resulting in adverse side effects. Our developed nano-formulation, DIP-FU-PPy NPs, showed highly targeted accumulation toward inflamed endothelial cells (Fig. 3e, DIP signal) compared to that in the normal cell group (without LPS) treated with DIP-FU-PPy NPs. The fluorescence intensity of LPS-treated cells treated with DIP-FU-PPy NPs was approximately 2.38-fold higher than that of non-LPS-treated cells. FU allowed the DIP-FU-PPy NPs to attach to the plasma membrane of target LPS-induced cells, according to a magnified image (Fig. 3i). Thus, this nano-development could achieve further therapeutic efficacy in thrombotic conditions.

#### In vivo thrombosis induction and in vivo vascular fluorescent assessment

**Fig. 4:**
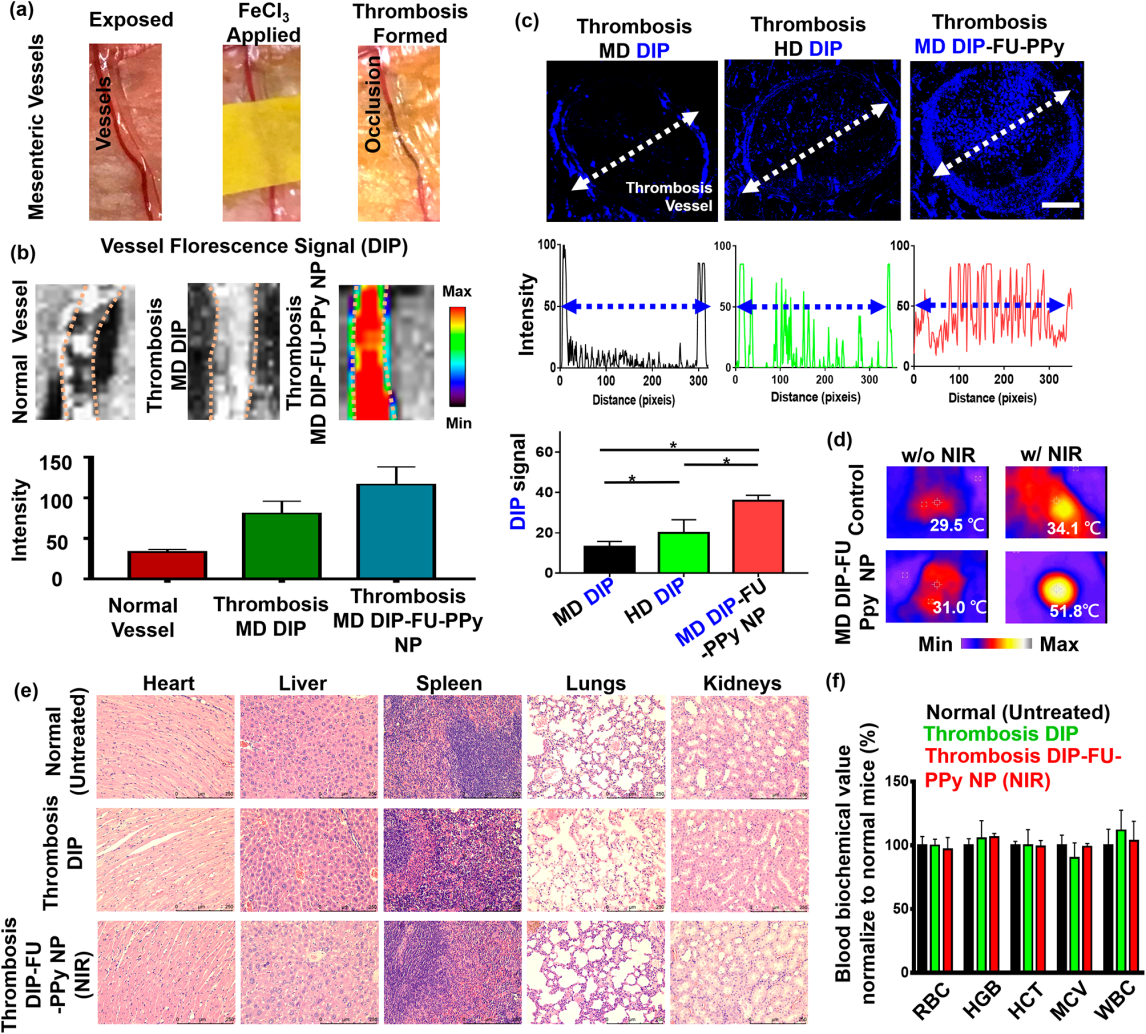
(**a**) Photographic observations demonstrating the creation of a thrombosis model using a ferric chloride filter paper method (filter paper was soaked in a 35% FeCl_3_-6H_2_O solution and positioned on the identified vessel). (**b**) IVIS (IVIS Lumina III XRMS) data showing a qualitative image and qualitative analysis of fluorescent dipyridamole (DIP) levels along the dotted lines of different formulations (moderate dose (MD) DIP or MD DIP-fucoidan (FU)-polypyrrole (PPy) nanoparticles (NPs)). (**c**) Fluorescence intensity of DIP in clot in fluorescence images of thrombus sites as analyzed by ImageJ software. The biodistribution results indicated that thrombi treated with free-DIP were unable to explicitly accumulate DIP or allow it to penetrate at the thrombus site. The thrombus site of the group given DIP-FU-PPy NPs exhibited deeper penetration and greater accumulation of DIP. (**d**) Thermal imaging (A-BF, RX300) showing that locally applied NIR (808 nm, 2.0 W/cm^2^ for 10 min) could induce hyperthermia in the group that received DIP-FU PPy NPs. (**e**) Representative photomicrographs of H&E-stained soft tissues (liver, heart, lungs, spleen, and kidneys) after treatment with DIP-FU-PPy NPs. H&E-stained microscopic and hematological data indicated that treatment with DIP-FU-PPy NP had in vivo biological safety (scale bar: 250 μm). (**f**) Hematological studies and biochemical indexes (red blood cells (RBCs), hemoglobin (HGB), hematocrit (HCT), mean corpuscular volume (MCV), and white blood cells (WBCs)) assessed using a blood serum hematological analyzer (Procyte Dx, IDEXX Laboratories). Experimental results are presented as the mean ± SD (*n* = 6). * *p* < 0.05; NS (non-significant) at *p* > 0.05). (Moderate dose (MD) DIP equivalent: 3 mg/kg body weight; high dose (HD) DIP equivalent: 5 mg/kg body weight)

To better understand the enhanced efficacy of DIP-FU-PPy NPs in the ferric chloride-induced mouse mesenteric thrombus model (Fig. 4a), thrombus targeting and the penetration ability were examined by IVIS and fluorescence microscopic imaging assays. As shown in Fig. 4b, after administration of DIP-FU-PPy NPs, a strong DIP fluorescent signal was detected at the thrombus site, indicative of its effectual thrombus targeting because of selective binding to P-selectin around the surface of activated platelets at the thrombus site. However, only weak fluorescent signals were detected for the vessel in the free DIP group with expression in the thrombus or the group in normal mice (Fig. 4b).

Figure 4c shows that for the free DIP-treated group (MD, 3 mg/kg), very weak fluorescence was detected only at the edge of the thrombosis, suggesting a negligible affinity toward the thrombotic site and inefficiency in penetrating the vessel structure and the thrombus center. The HD of free DIP (5 mg/kg) demonstrated slightly enhanced blue fluorescent signals at the edge of the thrombus, with a low signal within its core (Fig. 4c). Treatment with DIP-FU-PPy NPs (MD, 3 mg/kg) caused a remarkably greater and deeper-blue fluorescent signal across the thrombus from the edge to the core compared to that of free-DIP (Fig. 4c). According to earlier findings [58], a FU-functional supramolecular DDS was reported to favor deeper tissue penetration into vascular lesions, with the hypothesis that such nanoscale carriers possess increased hemodynamic behavior that to some extent, mimic the migration of leukocytes to vascular lesions. The deep penetration of the formulations may have been promoted by the nano-size of the prepared carriers. As previously reported, a network of fibrin forms pores smaller than 1 μm in thrombus clots [59], and the nanosized carrier range can penetrate deep into the interior of a clot. These nanocarriers can release drugs in response to NIR inside the clot and facilitate intra-clot lysis, which is consistent with the higher thrombolytic activity observed below. This could be attributed to the effective thrombus targeting of DIP-FU-PPy NPs owing to the enhanced penetration of nanosized particles along the systemic circulation with selective binding through P-selectin interactions. These in turn facilitated the effective penetration of therapeutics toward the thrombus site and triggered the release of DIP therein.

#### In vivo efficacy, biodistribution, and biosafety studies

Hyperthermia can be achieved once the region of the thrombotic site/vessel is irradiated with NIR, implying that precise and sufficient accumulation of NIR-photothermal switchable DIP-FU-PPy NPs occurred in vivo (Fig. 4d). The histological data (Figure S2) and previously published findings [60–62] indicated that a NIR or mild photothermal hyperthermic effect does not cause vessel injury. For in vivo safety assessments, H&E staining revealed that DIP-FU-PPy-NP treatment, similar to the other treatment groups (untreated healthy control and free DIP), did not cause inflammation or notable toxicity in major tissues (liver, heart, spleen, kidneys, or lungs, Fig. 4e). The hematological data proved that treatment with DIP-FU-PPy NPs (MD, 3 mg/kg) resulted in negligible changes in blood indices (Fig. 4f). The major biochemical indices were similar to normal levels in healthy animals. These findings suggest that DIP-FU-PPy NPs are biologically safe in vivo. Numerous investigations have provided biocompatible NIR technology at about 2 W/cm^2^ for precision treatment in several biomedical uses [63, 64]. Thus, the use of an NIR source can be applied in biomedicine.

Once the nanomedicine is administered in vivo, a host of inherent immunological mechanisms begin to identify and collect these foreign particles/substances and direct them toward the major elimination pathways from the body. There is thus always competition between the desired distribution of nanomedicines in specific tissues and the highly bioactive clearance mechanisms. The extent and distribution pattern of a nanomedicine in different tissues and organs, during a clinical therapeutic or diagnostic application, are usually considered the biodistribution. The rate of their recognition and removal by the immune system, excretion, and metabolism from the body is generally referred to as pharmacokinetics. Determining the biodistribution and pharmacokinetic characteristics is vital to augmenting the expected functionality of a nanomedicine in any selected region or tissue of the body and minimizing toxicological side effects because of any undesirable pharmacokinetic behaviors or biodistributions [65]. To the best of our knowledge, studies conducted to investigate PPy-based nanocarrier metabolic and biodistribution behavior are rare.

**Fig. 5:**
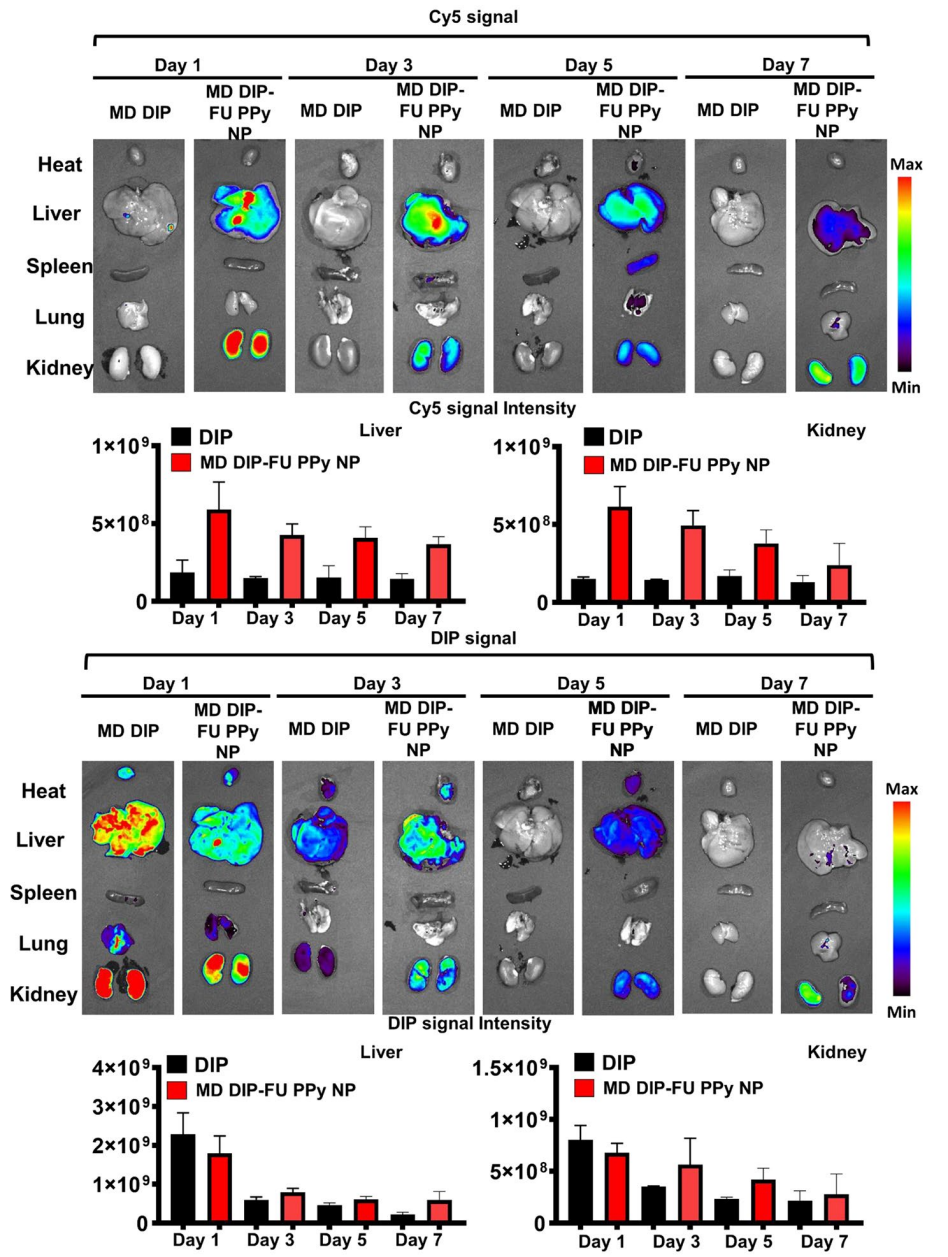
In vivo imaging and biodistribution study. Real-time in vivo fluorescence images of free-form dipyridamole (DIP) and DIP-fucoidan (FU)-polypyrrole (PPy) nanoparticles (NPs) conjugated with Cy5 in test mice at 1, 3, 5 and 7 days, assessed by IVIS. Semiquantitative fluorescence results of kidney and liver tissues. The representative organs in each group of IVIS images (for DIP or Cy5 distribution) may be either the same or different in vivo

To study the metabolic effect behaviors and biodistribution of DIP-FU-PPy NPs, test mice were administered free DIP or DIP-FU-PPy NPs. As shown in Fig. 5, free DIP was quickly eliminated from body at 1 day, and most DIP signals were found in the liver and kidneys. On the contrary, relatively low signals in the liver and kidneys were found in DIP-FU-PPy NP-treated mice. The prolonged circulation was attributed to the small particle size, colloidal stability, and negative charge of DIP-FU-PPy NPs. It seemed that DIP-FU-PPy NPs exhibited significant retention capabilities.

DIP-FU-PPy NPs showed an absolute advantage over free DIP in the battle of thrombus targeting, avoidance of clearance, and retention effects, which was evidenced by fluorescence images. More importantly, the fluorescence of DIP and the Cy5 carrier in excised liver and kidneys lasted as long as 7 days and was gradually eliminated through the kidneys (Fig. 5). The higher drug targeting, retention, eventual degradation, and elimination abilities may result in better biocompatible, therapeutic, and metabolic responses. These findings suggested that DIP-FU-PPy NPs showed good nanosystem circulation, biosafety, and enhanced bioavailability, retention, and metabolic abilities.

#### In vivo analysis on the effectiveness of P-selectin targeting/inhibition, its antithrombotic efficacy, and possible effects

**Fig. 6:**
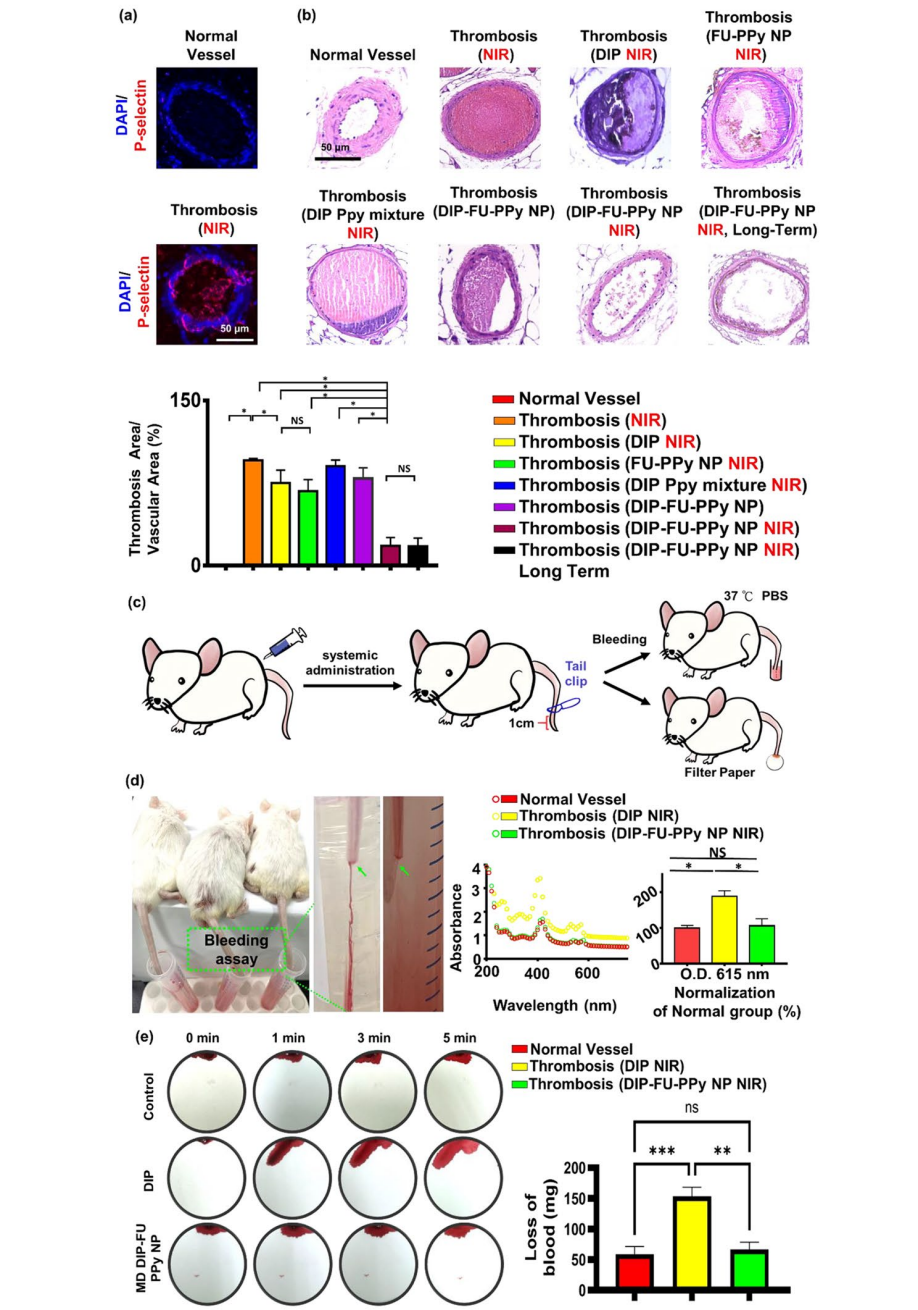
(**a**) In vivo histological results of fluorescent microscopy showing the expression of P-selectin in vessels. (**b**) In vivo results of light microscopy showing vascular H&E histological changes (scale bar: 50 μm), thrombus treatment, and prevention of thrombus regeneration in animals treated with different formulations. The antithrombotic efficacy was analyzed using the following method: (histologically microscopic vascular clot area/lumen area) × 100%. The calculated areas or microscopic fluorescence intensities were analyzed using ImageJ software. (**c**) Schematic illustration depicting the tail bleeding test. (**d**) For the tail bleeding test, after administration and treatment with dipyridamole (DIP)-fucoidan (FU)-polypyrrole (PPy) nanoparticles (NPs) or the same amount of DIP (moderate dose (MD), 3 mg/kg body weight), the distal 1-cm section of a mouse tail was cut off using a scalpel and submerged in PBS prewarmed to 37 °C. Animals were observed for 30 min. To calculate the bleeding quantity, the bleeding extent was assessed based on the hemoglobin level in the PBS solution by determining the absorbance spectra of hemoglobin. (**e**) For another bleeding test, filter paper was used to absorb blood from the bleeding liver of test animals. Blood-diffused areas on the filter paper were measured using ImageJ software. Microscopic and biochemical data confirmed that the DIP-FU-PPy NPs possessed antithrombotic efficacy and avoided clot reformation and bleeding risk in vivo. Experimental results are presented as the mean ± SD (*n* = 6). (* *p* < 0.05; NS (non-significant) *p* > 0.05)

P-Selectin is expressed on the surface of activated platelets and is a thromboinflammatory biomolecule engaged in platelet aggregation and activation. In addition, FU has a high affinity for P-selectin and is, therefore, suitable for specific therapeutic delivery and diagnoses of bioactivities associated with P-selectin expression, such as pathological thrombotic organs. Data obtained from a microscopic evaluation of vessel thrombosis after ferric chloride exposure are shown in Fig. 6a. Exposure to ferric chloride caused large, compact thrombi, which closely resembled the thrombus phenotype. Ferric chloride-induced thrombus formation and increased in vivo P-selectin expression are known to be connected [66]. An immunofluorescent histological analysis revealed that P-selectin was highly expressed in thrombotic vascular clots (higher than that in the control group; Fig. 6a). The FU nanocarrier specifically accumulated in thrombotic clots. The vessel surface at the ferric-treated vascular thrombosis site expressed P-selectin, leading to fibrin clot formation, as observed in our histological data (Fig. 6a).

As shown in Fig. 6b, a plugged thrombus was clearly retained in the control group (thrombosis + NIR). Treatment with free DIP (MD, 3 mg/kg BW) resulted in a slight decrease in the thrombus size. It was found that the retained thrombus after the free-DIP treatment under NIR irradiation had a smooth surface, caused by poor penetration (Fig. 6b), and the resultant antithrombotic bioactivity was limited to the surface of the thrombus. In contrast, treatment with DIP-FU-PPy NPs (MD, 3 mg/kg BW) with NIR irradiation led to significant thrombus dissolution (with only around 17% of the clot area remaining), further confirming that the antithrombotic efficacy was substantially higher than that of the control (with around 95.73% of the clot area remaining) and free-DIP groups (with around 75.03% of the clot area remaining). Histological data from test animals administered DIP alone with subsequent NIR treatment showed an anticlotting effect at the induced thrombus site. This finding suggests that a considerable dose of DIP is required to achieve an anticlotting effect. Test animals administered the mixed formulation of DIP and PPy without FU but with NIR irradiation showed negligible thrombus dissolution (with around 90% of the clot area remaining). Test animals administered DIP-FU-PPy NPs without NIR irradiation revealed a limited thrombus dissolution efficacy (with around 79.18% of the clot area remaining, Fig. 6b). Test animals administered FU-PPy NPs with NIR irradiation revealed a partial thrombus dissolution outcome (with 67% of the clot area remaining). These findings suggested that hyperthermia, FU and DIP were involved in the antithrombotic action. Therefore, animals administered FU-based NPs with subsequent NIR treatment exhibited considerable reductions in clots in the vascular lumen compared to thrombotic animals administered systemic DIP or thrombosis alone with subsequent NIR. This implies that the administered DIP-FU-PPy-NPs specifically accumulated at the FeCl3-induced P-selectin-expressing thrombotic site to facilitate the anticlotting efficacy of the combined therapy (DIP and photothermal ablation) (Fig. 6b).

Histological and quantitative results showed that after animals were administered DIP-FU-PPy-NPs and subjected to sequential irradiation with NIR, regeneration of the thrombosis was prevented for 1 day (Fig. 6b). The superior antithrombotic efficiency and prevention of thrombus regeneration were attributed to augmentation of the antithrombotic effectiveness of the NIR-triggered release of DIP and hyperthermia. The incorporation of FU-decorated P-selectin-targeting NPs precisely delivered these therapeutics to the thrombus clot.

A rodent tail-bleeding assay was used to assess the risk of hemorrhaging with DIP-FU-PPy-NPs (Fig. 6c, d). In this assay, a greater tail-bleeding volume was associated with a higher hemorrhage risk [67]. As shown in Fig. 6d, the distal 1-cm segment of the tail of a mouse was excised using a medical scalpel to induce bleeding, and the extent of bleeding was determined at 1 h after administration of free-DIP (NIR) or DIP-FU-PPy-NPs (NIR) (MD, 3 mg/kg BW). The bleeding quantity induced with free DIP (MD, 3 mg/kg BW) remarkably increased compared to that induced by DIP-FU-PPy-NPs. As shown in Fig. 6d, the bleeding extent assessed by the hemoglobin absorbance assay in animals treated with free DIP was significantly greater than that in the DIP-FU-PPy-NP-treated group (as the absorbance increased around 1.78-fold relative to that in the DIP-FU-PPy-NP-treated group). A comparison between the normal animals and thrombotic animals treated with DIP-FU-PPy NPs and NIR yielded no statistically significant differences in the bleeding assay results (Fig. 6d). This suggests that FU-modified NPs localized the therapeutics toward the thrombotic region and then initiated precise delivery of DIP upon NIR treatment to initiate site-specific therapeutic/photothermal thrombolytic effects with great therapeutic efficiency. Thus, adverse events due to therapeutic-generated hemorrhaging in normal organs were avoided. The extent of bleeding from the liver as assessed using blood absorption onto filter paper in animals treated with free DIP (NIR) was significantly greater than that in the DIP-FU-PPy-NP-treated group (blood absorption onto filter paper increased about 2.3-fold relative to the DIP-FU-PPy-NP-treated group) (Fig. 6e). These findings indicated that DIP-FU-PPy-NPs can remarkably reduce nonspecific hemorrhagic side effects of free DIP used for thrombus treatment. As previously reported, the clinical use of DIP in thrombotic treatment increases the risk of severe bleeding [68]. Massive hemorrhaging attributed to the uncontrolled release of thrombolytic drugs is a crucial issue in thrombus treatment in clinical practice [69]. The enrichment provided by DIP-FU-PPy-NPs resulted in augmented DIP delivery, increased thrombolytic efficacy, and a reduced risk of hemorrhaging.

**Fig. 7:**
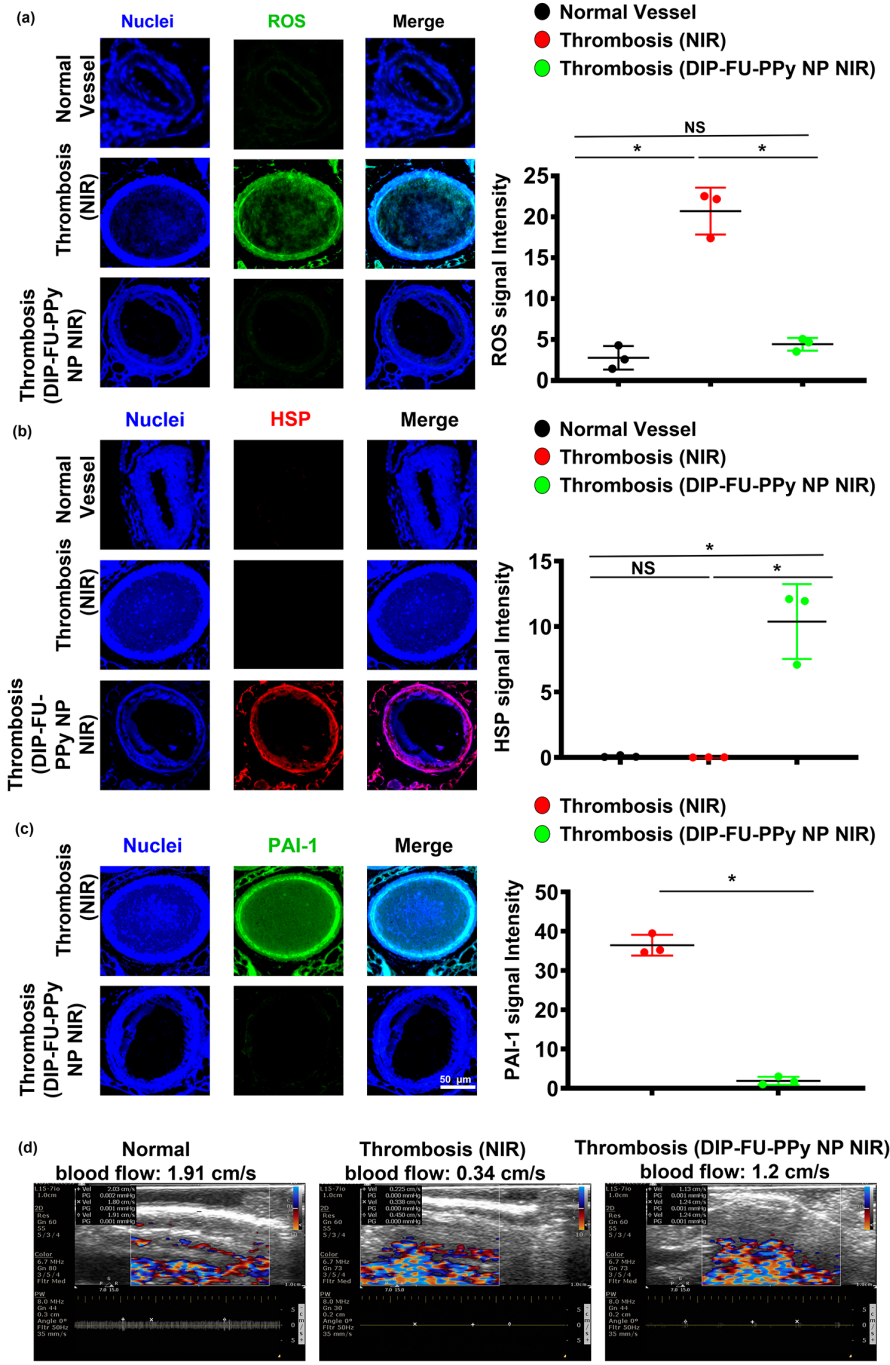
In vivo therapeutic mechanism. Microscopic data and semiquantitative fluorescence results of vascular (**a**) reactive oxygen species (ROS), (**b**) anti-heat shock protein (HSP), and (**c**) anti-plasminogen activator inhibitor (PAI)-1 levels of test mice (normal, thrombotic animals that received near infrared (NIR) irradiation, and thrombotic animals that received dipyridamole (DIP)-fucoidan (FU)-polypyrrole (PPy) plus NIR). (**d**) Ultrasonic data of vascular blood flow velocities of test mice (normal, thrombotic animals that received NIR, and thrombotic animals that received DIP-FU-PPy plus NIR). * *p* < 0.05; NS (non-significant) at *p* > 0.05)

Oxidative stress is a vital event under both pathological and physiological situations. As no single genetic biomarker or test precisely predicts the extent of vascular health, phenotyping for biomarkers of inflammation should identify individuals at risk for vascular illnesses. Reactive oxygen species (ROS) are important mediators of signaling pathways that cause vascular inflammation in thromboses [70]. In this study, we quantified oxidative stress by measuring cellular ROS using DCFH-DA staining of vascular tissues. Microscopic data showed that thrombotic vessels had the greatest ROS levels, compared to normal vessels of healthy animals (Fig. 7a). Various animal thrombosis models support the notion that enhanced vascular ROS production from a dysfunctional mitochondrial respiratory chain plays a causative role in thromboses and other vascular diseases [70]. Anti-inflammatory approaches can prevent thrombotic events [71]. The vascular ROS level of thrombotic animals that received DIP-FU-PPy NPs plus NIR after long-term treatment showed similar outcomes as normal vessels. The microscopic data indicated that the vascular HSP expression level of thrombotic animals that received DIP-FU-PPy NPs plus NIR after long-term treatment was higher than those of the untreated thrombus and normal control groups (Fig. 7b). According to previously published findings, hyperthermia-induced HSP prevents or arrests inflammatory damage [72]. Along with the delivered anticoagulant, DIP, the phototherapeutic DIP-FU-PPy NPs produced a vascular anti-inflammatory effect plus HSP expression which was involved with restoration of vascular damage and avoidance of rethrombosis. Microscopic data indicated that the expression level of vascular plasminogen activator inhibitor (PAI)-1 of thrombotic animals that received DIP-FU-PPy NPs plus NIR after treatment was lower than that of the untreated thrombus group (Fig. 7c). We verified vascular blood flow velocities with different treatments (Fig. 7d). The vascular blood flow velocity was measured using ultrasound. Compared to the normal control group, the vascular blood flow velocity at the thrombotic site was reduced, while the vascular blood flow velocity in the DIP-FU-PPy NPs plus NIR treatment group was basically unchanged. These findings revealed that the fibrin clot was lysed due to dissolution of the thrombus after DIP-FU-PPy NPs plus NIR treatment, and the vascular blood flow velocity had returned to normal.

In previous studies [61, 73], we developed PPy-based carrier systems using the NIR-photothermal effect for thrombus treatment. A fibrin-targeting peptide-engineered nanoassembly of photothermal agents and the antiplatelet, ticagrelor, was prepared for thrombus-targeting delivery with a high thrombolysis efficacy [60]. However, the lack of the ability to avoid clot recurrence, bleeding risk, and knowledge regarding the underlying mechanisms of the inhibitory effect of rethrombosis, restoration of damaged vessels, biodistribution, and carrier degradability limited its performance in thrombus management. In this study, we designed DIP-FU-PPy-NPs that possessed thrombus-homing capabilities and NIR-phototherapeutic properties (refer to Figs. 1, 2 and 3). DIP-FU-PPy NPs demonstrated an amplified thrombolysis efficacy, and the ability to prevent clot recurrence, minimize the bleeding risk, and inhibit rethrombosis through well-understood mechanisms (refer to Figs. 4, 5, 6 and 7). Furthermore, the DIP-FU-PPy NPs promoted restoration of damaged vessels, exhibited favorable biodistribution, and demonstrated carrier degradability.

Precise medicine necessitates accurate early diagnoses and remote noninvasive and individualized proper treatments, and phototheranostics are considered to be the frontier for providing safe and quick disease localization and effective therapies. Semiconducting nanomaterials, such as organically conjugated polymers, small molecules, and inorganic semiconductors with photonic features, are being explored in medical imaging and phototherapy (photodynamic, photothermal, and photo-controlled combination therapies). In practical clinical applications, semiconducting organic materials are favored over inorganic materials for phototheranostics owing to their natural metabolism and biocompatibility. The supramolecular self-assembly technique is thought to be a significant means of preparing multifunctional and organic detachable phototheranostics with supramolecular interactions, such as hydrophobic effects, electrostatic and π–π interactions, and hydrogen bonding, which are dynamic and noncovalent.

In clinical experience, laser phototherapy was proven to be safe, effective, and valid for thrombolysis [74–77]. Biocompatible NIR technology takes advantage of the low absorption spectrum of biological tissues in the NIR range (650–950 nm) and can penetrate deep tissues. Studies confirmed that NIR plays a vital role particularly in clinical applications where deep tissue organ penetration (for tendons, bones, ligaments and cartilages) is required [78]. In clinical practice, NIR laser ablation has been well reported as a minimally invasive method to treat thrombotic diseases, such as varices of the lower extremities [79]. Due to strong optical absorption by thrombi, lasers can be used to dissolve clots and, therefore, can serve as an emerging option in patients with complicated thromboses who fail to respond to thrombolytic drugs and exhibit thrombotic lesions that are deemed unsuitable for standard balloon angioplasty [75]. Targeted nano-DDSs have shown great potential as clinically translatable phototheranostic agents for treating thromboembolisms. Overall, there is a strong prerequisite for further improvements and development of photo-thrombolysis, leading to its clinical translation [18]. As to future prospects, the use of NIR combined with minimally invasive angiographic technologies can realize phototherapy in deep vascular lesions [80].

## Conclusions

A practical co-delivery strategy was created in this research to intend to address several difficulties in thromboembolism therapy, including (i) insufficient active substance accumulation in thrombotic lesions, (ii) uncontrolled release of medications, (iii) insufficient permeability into deep thrombi, and (iv) low antithrombotic efficacy with bleeding vulnerability. It was interesting to observe how PPy, FU, and DIP co-assembled into stable nano-vehicles (DIP-FU-PPy NPs) that were stable in physiological condition. The multipurpose actions of DIP-FU-PPy NPs were as expected, including high DIP-loading efficiency, facile fabrication procedures, good thrombus-targeted biodistribution, remote NIR-photothermal responsiveness, and efficient thrombolytic activity. In a ferric chloride-induced mouse model of thrombi, the effect promoted substantial NIR-photothermal-augmented in vivo thrombus therapeutic effectiveness by causing thrombotic clots to disintegrate.

### Electronic supplementary material

Below is the link to the electronic supplementary material.

Supplementary Material 1

## Data Availability

The datasets used in this study are available from the corresponding author upon reasonable request.
