# Supplementary material for: P-Selectin mediates targeting of a self-assembling phototherapeutic nanovehicle enclosing dipyridamole for managing thromboses
Source: J Nanobiotechnology. 2023 Aug 8;21:260. doi: 10.1186/s12951-023-02018-7 (PMC10408148; doi:10.1186/s12951-023-02018-7)
Supplement: Supplementary file 1 — Supplementary Material 1 [file 12951_2023_2018_MOESM1_ESM.docx]

**Supporting information**


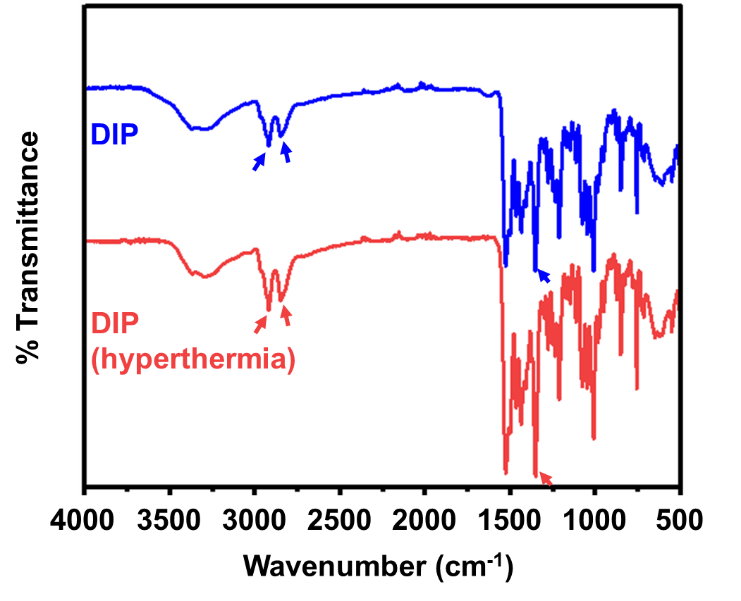


Fig. S1: FTIR data of DIP without or with hyperthermia treatment


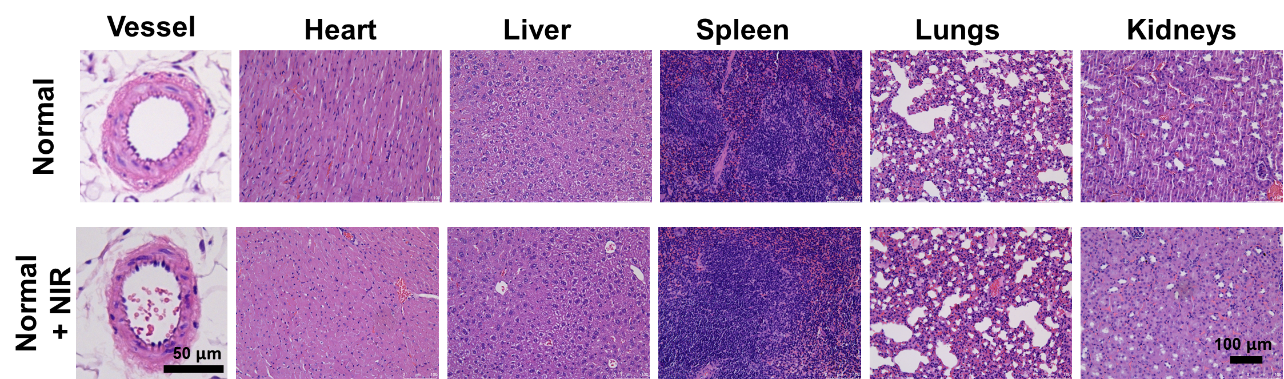


Fig. S2: Histological data of soft tissues (vessel, heart, liver, spleen, lung and kidney) of normal health and normal health plus NIR groups
